# Shape and Size Variability of the Gynostemium in *Epipactis helleborine* (L.) Crantz (Orchidaceae)

**DOI:** 10.3390/biology14030241

**Published:** 2025-02-27

**Authors:** Zbigniew Łobas, Anna Jakubska-Busse

**Affiliations:** Department of Botany, Faculty of Biological Sciences, University of Wroclaw, 50-328 Wroclaw, Poland; anna.jakubska-busse@uwr.edu.pl

**Keywords:** *Epipactis helleborine*, geometric morphometric methods (GMMs), gynostemium shape and size, Orchidaceae

## Abstract

*Epipactis helleborine* (L.) Crantz is considered a challenging and phenotypically difficult species to identify due to its wide range of morphological variability. This variability is mainly observed in the perianth parts but also extends to the gynostemium structure, which has so far been considered one of the most useful diagnostic characteristics. As a result, a simple graphic illustrating the structural pattern of gynostemium morphology has appeared in 10 different forms in available European taxonomic keys, which significantly complicates the identification of this species. A total of 122 flowers of *E*. *helleborine* were collected from four natural populations in the Lower Silesia region (Poland) between 2017 and 2019 and analysed for gynostemium morphological variation. Geometric morphometric analyses, including Procrustes ANOVA, PCA, and CVA, were used to examine gynostemium shape, with statistical tests assessing variation in size and stigma inclination angle among populations, individual plants (ramets), and years of research. Statistical analysis revealed significant positive correlations between gynostemium width and height, with significant variation in size and angle of stigma inclination, primarily driven by population, while ramet and year of research had a lesser impact. Geometric morphometric analyses indicated significant population-level variation in gynostemium shape, with principal component analysis identifying the ventral view as the most informative for discriminating these differences. The first two principal components explained the major shape variation, and canonical variate analysis confirmed that this view is most important for species identification.

## Simple Summary

Morphological variability of the gynostemium structure in *Epipactis helleborine* (L.) Crantz complicates species identification. A total of 122 flowers of *E*. *helleborine* were collected, prepared, and analysed for gynostemium morphological variation. Geometric morphometric methods (GMMs) and statistical tests were used to assess variation in the shape, size, and stigma inclination angle among populations, individual plants (ramets), and years of research. The results indicate that most variations in gynostemium shape and size show a correlation with population, with relatively little variation within populations or between research years. Both morphometric parameters and the overall structural pattern of gynostemium morphology, including dorsal, frontal, ventral, and right lateral views, should be considered for taxonomic identification of *E*. *helleborine*.

## 1. Introduction

Plant morphology has historically been the main driving force in developing traditional taxonomy. The foundations of modern botanical nomenclature were laid by prominent taxonomists, including Linnaeus, who developed plant classification systems based on morphological characteristics. Despite the widespread availability of ecological, geographical, and genetic data for taxonomic research, botanists still largely rely on external morphology to identify and classify plants. This is because morphological characteristics are relatively easy to describe and compare, making them important in determining relationships not only between species but also within higher taxa, such as genera or families [1]. However, the morphological variability observed in numerous plant species, including orchids, makes it challenging to identify useful diagnostic characteristics. This is particularly evident when attempting to establish reliable diagnostic criteria [2].

Researchers studying the morphological variability in the genus *Epipactis* Zinn, 1757 have long focused on the identification of taxonomically useful, unambiguous diagnostic characteristics. The results of decades of research have culminated in available taxonomic keys, which have so far include characteristics, such as: height, colour, and hairiness of the stem; size, shape, and colour of leaves; the ratio of leaf length to internode length; type of leaf arrangement; size, shape, and angle of inclination of papillary cells present along the veins and/or at the edge of the leaf blade; size, shape, and colour of individual parts of perianth, especially the lip; shape of the transition between the apical and basal parts of the lip; and the gynostemium structure (i.e., [3,4,5,6,7,8,9,10,11,12,13,14,15,16,17,18]). Although these morphological characteristics have proved useful in distinguishing species, their wide range of variation can often complicate the identification process, particularly in populations with high levels of phenotypic plasticity [19,20,21,22,23,24].

None of the morphological characteristics mentioned previously has received as much recent attention as the gynostemium structure, which is now considered one of the most useful diagnostic characteristics for distinguishing taxa within the family Orchidaceae [25,26,27,28,29,30,31]. The gynostemium is a centrally located generative floral organ that, in *Epipactis* species, results from the fusion of the single median outer stamen and the stigmas [25,32]. Compared with other parts of the orchid plant, the morphology of the gynostemium is clearly distinguished by its consistent and repeatable structural pattern, especially within closely related taxa [26,33]. However, unpublished research indicates that this structure, like other floral organs, can exhibit considerable morphological variability, supported by field observations such as those on the Eurasian orchid species *Epipactis helleborine* (L.) Crantz, 1769 [34].

**Figure 1:**
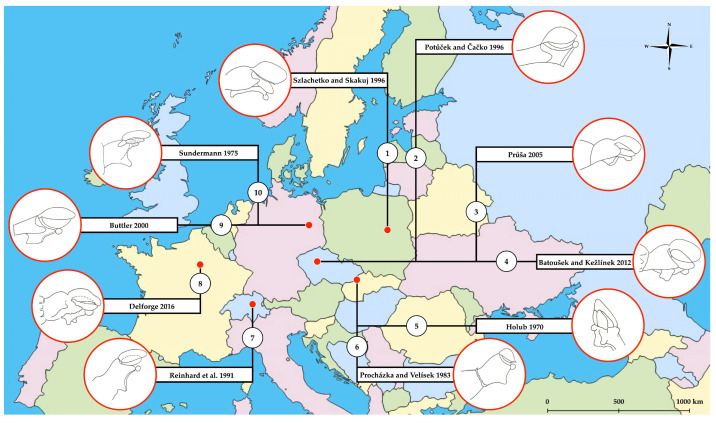
The graphic representations of the structural pattern of the gynostemium morphology of *Epipactis helleborine* from various European taxonomic keys. According to [3,4,5,6,8,9,11,12,15,35].

Numerous European studies devoted to the taxonomy of *E*. *helleborine* have been supplemented with a simple graphic illustrating the structural pattern of gynostemium morphology (Figure 1). The inconsistency in the presentation of this taxonomically useful floral organ can lead to confusion, particularly for those attempting to identify *E*. *helleborine* under field conditions. As a result, the varying forms of graphical depictions of the gynostemium significantly limit their reliability as a tool for distinguishing *E*. *helleborine* from other allogamous species within the genus [3,4,5,6,8,9,11,12,15,35].

The aim of our study was to investigate the morphological variability of the gynostemium in four different populations by measuring its size, assessing the angle of inclination of the stigma, and, for the first time, performing geometric morphometric analyses to examine its shape. Based on these studies, the morphometric parameters have been established, and detailed graphical illustrations of the morphology of the *E*. *helleborine* gynostemium have been produced, which provide a consistent and precise description of its general structure. The results not only help to facilitate the identification of *E*. *helleborine* but also offer new insights into its morphological variability, which may prove valuable for both scientific research and fieldwork in the future.

## 2. Materials and Methods

### 2.1. Study Area and Sampling Procedure

A total of 122 flowers were collected between 2017 and 2019 from four natural populations of *E*. *helleborine* in the Lower Silesia region (Poland) (Table 1). The selection of the sampling sites in this region was based on population size (a minimum of 100 flowering individuals) and the absence of other *Epipactis* species in the area, minimizing the risk of hybrid forms. Furthermore, limiting sampling sites to southern Poland was driven by the need to analyse fresh plant material, which could be reliably obtained due to the close proximity of the collection sites to the laboratory.

Flowers were hand-collected immediately after natural flower opening to ensure that only fully developed gynostemia were sampled for analysis. Individuals for sampling were selected randomly within each population to avoid any bias in the selection process. The collection of research material was carried out with the permission of the Regional Directorate for Environmental Protection in Wroclaw (permission nos.: WPN.6400.37.2017.IL, WPN.6400.25.2018.IL, WPN.6400.25.2019.MH).

Due to the structural changes that occur during the drying of plant material, including the complete flattening of the gynostemium structure in herbarium sheets, the use of dried flowers from herbarium collections was deemed unsuitable for this study. The drying process damages the gynostemium, thereby compromising the integrity of its important morphological characteristics and rendering it unsuitable for detailed geometric morphometric analyses. To ensure reliable results, only fully developed gynostemia were sampled from freshly opened flowers. This approach allows for an excellent preservation of all the morphological details that are necessary for the analyses carried out in this study.

**Figure 2:**
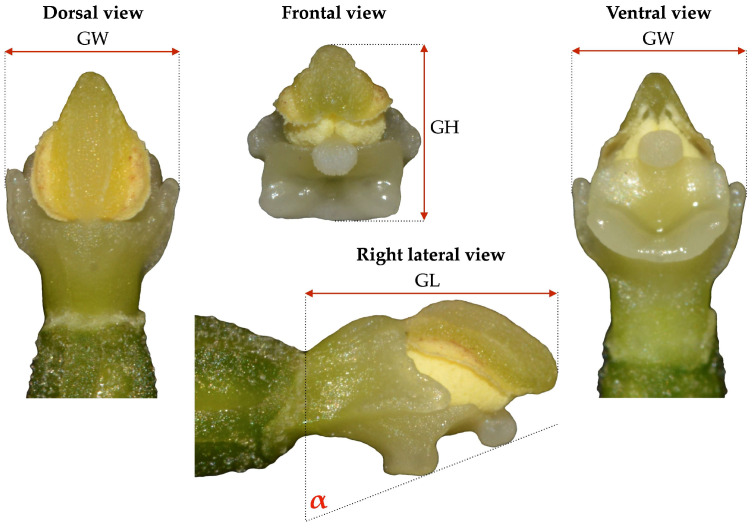
Morphometric measurements: GL—gynostemium length, GW—gynostemium width, GH—gynostemium height, α—stigma inclination angle.

The collected flowers were transported to the laboratory in isothermal containers, preserved with ice, for biological processing. The perianth parts of each flower were carefully removed with forceps, and the prepared gynostemium was then photographed using a Nikon D3200 camera (Nikon Corporation, Tokyo, Japan) equipped with a Tamron 90 mm F/2.8 macro lens from four standard viewing directions, i.e., dorsal, frontal, ventral, and right lateral. To minimize errors due to image distortion, all gynostemia were photographed at a fixed distance with an accurate 1 mm scale. The gynostemium was measured for length, width, and height, and the angle of inclination of its stigma was determined. The angle of inclination was measured between the outer edge of the stigma and a vertical line along the ovary apex (Figure 2). All measurements were performed using ImageJ version 1.53k [37] and are presented in the Supplementary Materials (see Table S1).

**Figure 3:**
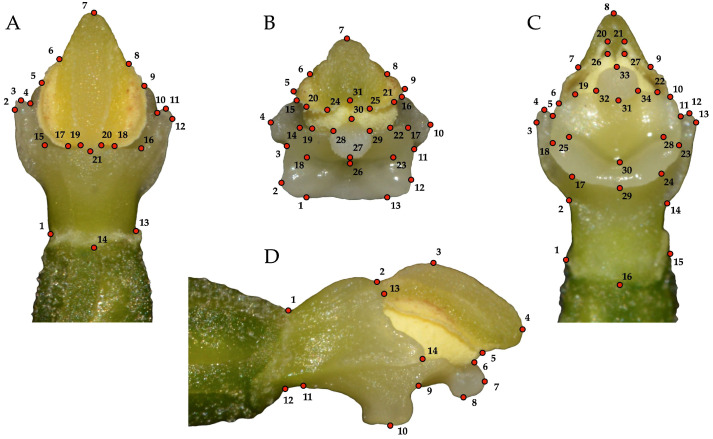
Digital illustration of the gynostemium morphology of *Epipactis helleborine* showing the selected landmarks for the dorsal (**A**), frontal (**B**), ventral (**C**), and right lateral (**D**) views.

JPEG images were used to create tps files using tpsUtil version 1.70 [38]. A total of 14 to 34 landmarks were defined within the gynostemium, depending on the views analysed. Landmarks were placed at the junctions between the different parts of the gynostemium (i.e., ovary, filament, connective, theca, staminodium, clinandrium, pollinium, viscidium, stigma, rostellum, column part), as well as at the points of maximum elongation (Figure 3). Digitalisation of specimens was carried out by the same person (ZŁ) in tpsDig2 version 2.31 [38].

### 2.2. Geometric Morphometric Analyses of Gynostemium Shape Variation

Two-dimensional Cartesian coordinates of the entire sample of 122 gynostemium configurations, with four distinct landmark configurations for each of them, were imported into MorphoJ version 1.07a for further processing [39]. To eliminate variations due to differences in position, orientation, and scale, the raw landmark coordinates were aligned and superimposed using the software’s Procrustes Fit function, taking into account that the gynostemium exhibits object symmetry in dorsal, frontal, and ventral views. Given that the symmetric component usually explained over 80% of the total shape variation (i.e., 79.7% in the dorsal view, 82.7% in the frontal view, and 84.5% in the ventral view), the asymmetric component was excluded. The new Procrustes coordinates were then used for all geometric morphometric analyses [40,41]. Principal component analysis (PCA) was used to explore and summarise the patterns of gynostemium shape variation, while Procrustes ANOVA was used to partition the shape variation into different sources, such as variability between populations, within individual plants (ramets), and between survey years, and to explore the interactions between these factors. This analysis helped identify how each factor and its interactions contributed to the overall shape variation. Variability between populations, within ramets, and between years of research on gynostemium shape was examined using canonical variates analysis (CVA). The statistical significance of pairwise differences in mean shapes was analysed using permutation tests (10,000 rounds) with both the Mahalanobis distances and the Procrustes distances. A significance threshold of *p* < 0.05 was set, with results also considered significant at *p* < 0.01 and *p* < 0.001. Specifically, the Procrustes distances were used to evaluate the difference in shape after aligning the configurations, allowing for the detection of shape variation independent of differences in position, orientation, and scale. The Mahalanobis distances were calculated after applying the full Procrustes fit to the dataset to assess multivariate differences in shape between the groups. This approach ensures that only differences in shape are considered, excluding variations in position, orientation, and scale. In this case, the Mahalanobis distances are calculated directly on the Procrustes-aligned data, without accounting for any further covariance structure within the groups. These distances were used as a measure of shape variation among the groups, allowing for a more accurate comparison based solely on the shape components. A pair of superimposed wireframe graphs connecting the landmarks with straight lines for the starting (black lines) and target shapes (red lines) were then used to visualize shape differences for interpretation. For detailed results, please see the Supplementary Materials (Tables S2–S9, Figures S1–S13).

Based on the results of the generalised Procrustes analysis (GPA) of gynostemium shape, the mean positions of the landmarks were calculated. This allowed the creation of detailed graphical illustrations showing the general structural pattern of the gynostemium in all analysed views. Adobe Photoshop version 7.0 was used to create the illustrations.

### 2.3. Statistical Analysis of Gynostemium Size and Angle of Stigma Inclination

Descriptive statistics were used to characterise the morphological variability of the gynostemium. The mean, median, standard deviation, interquartile range, min–max range, and coefficient of variation were calculated for continuous variables, such as length, width, height, and angle of inclination of the stigma, and are available in the Supplementary Materials (Tables S10–S12). For nominal variables, such as population, ramet, and year of research, absolute and percentage frequency distributions were presented to describe the occurrence of each category. Box plots were used to compare the distribution of continuous variables among populations. To test for monotonic relationships between variables, Spearman’s rank correlation coefficient was used, which measures the monotonic dependency between the variables analysed. The strength of the correlations was determined according to Guilford’s criteria [42]. One-way ANOVA was used to compare differences in variances both between the groups and within the groups. In addition, relationships identified by one-way ANOVA were confirmed using linear mixed-effects model analysis, with random effects accounting for variability associated with the number of ramets. As the measurement results depend on the number of ramets, a linear mixed-effects model with random effects was used for the analysis. The reference values were the year 2017 and the population of Kotowice, against which the obtained results were compared. The fixed effects analysis provides model coefficients that indicate how the response variable changes with a unit increase in the explanatory variable. The random factor included in the model accounts for the variability of individual observations. An F-test and the Akaike Information Criterion (AIC) were used to determine the best-fitting model, taking into account possible interactions. Principal component analysis (PCA) was used to illustrate the relationships between continuous variables and individual gynostemium observations among populations. A significance threshold of *p* < 0.05 was set, with results also considered significant at *p* < 0.01 and *p* < 0.001. The normality of the distribution of morphological characteristics of the gynostemium was tested with the Shapiro–Wilk test. All statistical data analyses of gynostemium size and angle of stigma inclination were performed using R (version 4.0.2 and package Ismeans version 2.25) [43,44].

## 3. Results

### 3.1. Variations of Gynostemium Shape

The analysis of the covariance matrices revealed that the principal components (PCs) captured both symmetric shape variation (for the dorsal, frontal, and ventral views) and asymmetric shape variation (for the right lateral view). The principal component analysis (PCA) indicated the morphological differentiation among the studied populations, with particular emphasis on the ventral view, which proved to be most effective in distinguishing the populations.

In the ventral view, the first two principal components explained 51.61% of the total variance (PC1 = 34.25%, PC2 = 17.36%), indicating that these PCs captured the major shape differences between populations. These values were higher than those observed in the right lateral view (45.31%) and were comparable to those in the dorsal (57.72%) and frontal views (57.38%). The eigenvalues for PC1 and PC2 in the ventral view (λ = 0.00521850 and λ = 0.00264466, respectively) emphasised the significant contribution of key shape features to the observed population differentiation.

Following PCA, canonical variate analysis (CVA) showed clear separation across multiple views (dorsal, frontal, ventral, and right lateral), with robust test statistics, such as Goodall’s F and Pillai’s trace, confirming the significance of the observed differences. The most pronounced difference was found in the ventral view, where population differences were particularly distinct (Goodall’s F = 52.21, *p* < 0.0001; Pillai’s trace = 1.44, *p* < 0.0001). In contrast, the right lateral view showed significant variation primarily linked to differences between ramets (Goodall’s F = 13.81, *p* < 0.0001; Pillai’s trace = 6.67, *p* < 0.0001).

The first two canonical variates accounted for most of the variation in the data, with CV1 explaining the largest proportion of shape differences. For instance, in the ventral view, CV1 explained 48.45% of the total variation, clearly distinguishing the Milicz and Żelazno populations, while CV2 (38.74%) captured smaller differences among the remaining populations.

The Mahalanobis distance confirmed significant differences among populations across all analysed views. The greatest distances were recorded in the ventral view, particularly between the Kotowice and Milicz populations (Mahalanobis = 9.58, *p* < 0.0001), highlighting the prominence of shape differences in this view. In the right lateral view, the Mahalanobis distances were smaller but still statistically significant (e.g., Kotowice vs. Żelazno, Mahalanobis = 4.80, *p* < 0.0001).

The Procrustes distances also indicated significant shape differences among populations, though they were generally smaller than the Mahalanobis distances. For example, in the dorsal view, the Procrustes distance between Kotowice and Żelazno was 0.079 with a *p*-value of 0.0001, suggesting subtle but statistically significant shape discrepancies.

Temporal analysis revealed that the year of research significantly impacted morphological differences, particularly in the frontal and ventral views. In the frontal view, the Mahalanobis distance between the 2017 and 2019 datasets was 4.88 with a *p*-value of 0.0001, indicating substantial morphological changes over the study period.

Moreover, the analysis of ramets demonstrated significant variation within populations, with the most pronounced differences between ramets 1 and 8 in the ventral view (Mahalanobis = 9.21, *p* < 0.0001; Procrustes = 0.16, *p* < 0.0001), highlighting considerable intra-population variability.

Among all views, the ventral view proved to be the most effective for distinguishing populations and detecting morphological differences. The high Mahalanobis distances, alongside the significant Procrustes distances, test statistics, and PCA results, underscore that this view captures the most pronounced variability among populations, making it an invaluable tool for species or population identification in morphological studies.

### 3.2. Variations of the Gynostemium Size and Angle of Stigma Inclination

**Figure 4:**
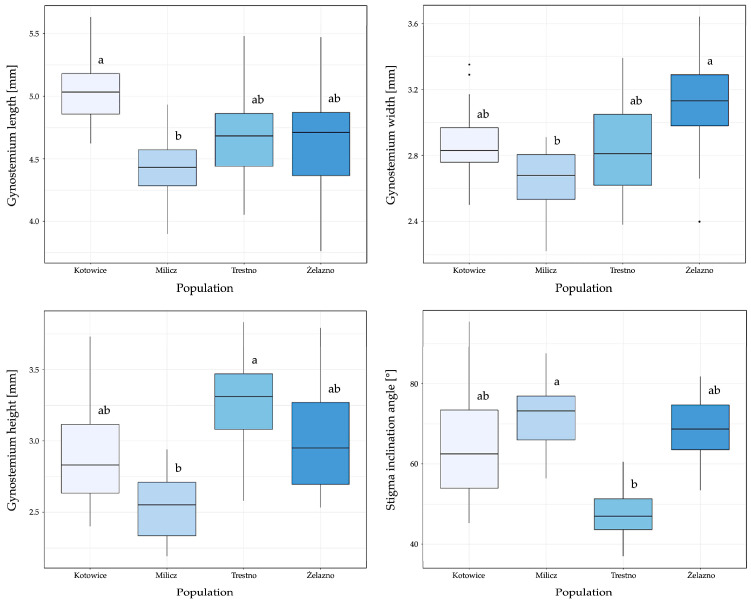
Box plots for analysed continuous variables: gynostemium length, gynostemium width, gynostemium height, and stigma inclination angle. Within each plot, the box represents the interquartile range (IQR), with the thick horizontal line indicating the median. The whiskers extend to 1.5 times the IQR beyond the first and third quartiles, and individual points outside the whiskers represent outliers. Sites with the same letter code are not significantly different, while sites with different letter codes are significantly different.

Statistical analysis revealed a significant positive correlation between the width and height of the gynostemium (Spearman’s coefficient r > 0.5, *p* < 0.001). Moderate positive correlations were also found between the length of the gynostemium and its width and height. When examining the relationship between gynostemium size and stigma inclination angle, a moderate negative correlation was observed only between height and stigma inclination angle. The mean gynostemium length ranged from 4.18 mm (ramet 10) to 5.09 mm (ramet 2), with coefficients of variation between 3.4% and 6.82%. The average width varied between 2.51 mm and 3.30 mm, with the greatest variability observed in ramet 3 (9.74%). Gynostemium height varied between 2.45 mm and 3.53 mm, with variability ranging from 3.58% (ramet 10) to 10.81% (ramet 7). The angle of stigma inclination varied between 46.87° and 77.15°, with the highest variation in ramet 5 (15.53%) and the lowest in ramet 11 (5.82%). In terms of population-level differences, the Kotowice population had the largest mean gynostemium length (5.03 mm) with the least variation (CV 4.82%). In contrast, the Żelazno population had the greatest variation in length (CV 8.62%) and the widest gynostemium (3.10 mm). The population of Trestno had the highest average height of the gynostemium (3.24 mm) and the smallest angle of inclination of the stigma (47.44°). The Milicz population had the smallest mean values for length, width, and height, but had the largest average stigma inclination angle (72.01°) with the lowest variation (CV 10.32%) (Figure 4).

**Figure 5:**
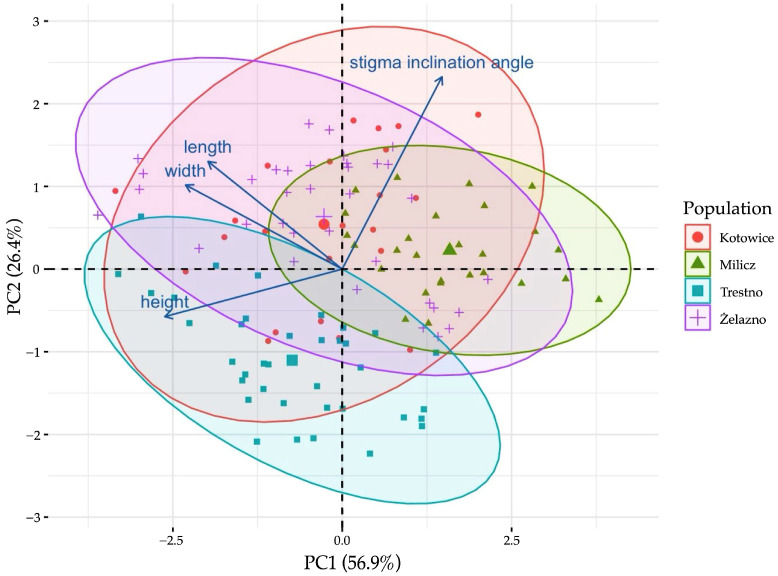
Biplot of the first two principal components from PCA for gynostemium morphological characteristics among four populations of *Epipactis helleborine*.

An analysis of the variation of gynostemium size across years of research showed that the average values remained similar over time, with slight increases observed. However, no significant year-to-year changes were observed for the stigma inclination angle. Population-level differences had a greater effect on gynostemium variability than intra-population differences, particularly for width and height, although statistical tests (ANOVA and mixed models) found no significant relationships between these variables and population. However, the variability of the stigma inclination angle showed significant differences between populations (*p* < 0.005). Analysis of variance confirmed that most of the variability in gynostemium morphological characters occurred between populations rather than within them, suggesting that population-level factors have a stronger influence on these characters (Figure 5).

### 3.3. General Structural Pattern of Gynostemium Morphology

**Figure 6:**
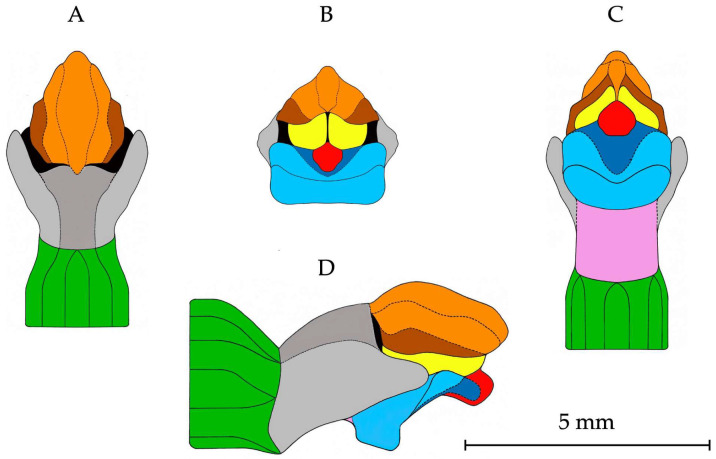
Graphical illustration of the structural pattern of the gynostemium in *Epipactis helleborine* showing detailed morphology for the dorsal (**A**), frontal (**B**), ventral (**C**), and right lateral (**D**) views. Various parts of gynostemium morphology are colour-marked: green—ovary, dark grey—filament, orange—connective, brown—theca, light grey—staminodium, black—clinandrium, yellow—pollinium, red—viscidium, light blue—stigma, dark blue—rostellum, violet—column part.

Graphical illustrations of the structural pattern of the gynostemium in *E*. *helleborine*, showing detailed morphology from four standard viewing directions, i.e., dorsal, frontal, ventral, and right lateral, have been produced from the geometric morphometric data and are presented in Figure 6. To facilitate their use in a manual identification process, the individual parts of the gynostemium have been precisely colour-marked. These figures represent the multi-view depiction of the gynostemium structure in *E*. *helleborine*.

## 4. Discussion

The morphology of the gynostemium plays an essential role in orchid taxonomy due to its relatively limited variability and the repeatability of its structural pattern [45,46,47]. It has been shown that its structure not only allows effective differentiation of higher taxonomic units, such as tribes, orders, or families, but also, in some cases, provides a valid basis for distinguishing and describing new species within a genus [48]. Furthermore, it is the most commonly presented diagnostic characteristic of the Orchidaceae family in graphical form and is included in the available taxonomic keys (e.g., [26]).

In the taxonomic identification of orchid species, a detailed analysis of the appearance of the gynostemium as a whole, as well as the details of its individual structural parts, is of great importance. This is particularly true for the rostellum, as shown by the work of Dressler [49], Kurzweil [47], Carnevali et al. [50], and Kobayashi and Arditti [51]. Equally important is the analysis of the structure of the clinandrium and the presence of the sticky viscidium produced by the rostellum [31].

While the taxonomic identification of individual genera within the Orchidaceae family based on the detailed morphology of the gynostemia observed under field conditions is not highly controversial, significant problems may arise when species are differentiated on the basis of herbarium material alone. Long-term storage of herbarium specimens has been shown to irreversibly damage or destroy most taxonomically useful characteristics, such as the overall structure of the shoot, colour of the stem, leaves or flowers [52,53]. Similarly, the gynostemium is often severely distorted or flattened by the drying process, which greatly reduces its taxonomic utility. In the case of the genus *Epipactis*, studies based solely on herbarium material, without analysis of freshly collected plant specimens, are prone to significant errors. The species identified in this way may in fact be different taxa producing gynostemia with a similar appearance [54,55]. This highlights the importance of conducting parallel taxonomic research on the Orchidaceae family based on freshly collected plant specimens [56].

The actual range of morphological variability in the gynostemium of specific *Epipactis* taxa has been poorly reported in the literature. This is despite the existence of representative research samples [7,16,17,18,57,58] that could provide the basis for such results. The gynostemium of autogamous and allogamous species of *Epipactis* differs significantly in structure [59]. The most useful diagnostic characteristic in the currently adopted classification system is the presence or absence of a viscidium [31]. The viscidium is directly responsible for attaching the pollinia to the body of potential pollinators. In most allogamous species, the viscidium is well developed and visible as a rounded globular mass of viscid fluid, covered by a thin membrane. At its back, a small pointed projection is attached to the pollinia [60].

However, it is important to consider the function of this part, which facilitates the pollination process by attaching to the insect body together with the pollinia. Therefore, the absence of a viscidium in a flower does not necessarily indicate that such a ramet belongs to an autogamous species. It is possible that the flower was previously visited by insects, which caused the viscidium to be removed mechanically. It is therefore crucial to examine gynostemia in freshly opened flowers or flower buds during taxonomic identification [34].

The comparison of the morphology of a fully developed gynostemium with a graphic illustration of its structural pattern is a commonly used method for the taxonomic identification of *Epipactis* species. It is worth noting that in the available European taxonomic keys, simple graphics illustrating the structural pattern of the gynostemium in *E*. *helleborine* have so far appeared in up to 10 significantly different forms [3,4,5,6,8,9,11,12,15,35]. Unfortunately, the graphics examined in our study often lack clear labels for the individual parts of the gynostemium, and in most cases even scale bars [3,4,5,6,8,12,15,35]. Furthermore, some of these figures [3,4,5,35] do not accurately depict characteristics that are critical to the cross-pollination process, such as the rostellum with an apical viscidium, which can make it difficult to identify the species based on these characteristics. In addition, the published graphics differ in their representation of the angle of stigma inclination (see Figure 1), which is strongly associated with the pollination strategy used.

The differences between the published graphics of the structural pattern of the gynostemium morphology of *E*. *helleborine* can be attributed to two factors. Firstly, it is notable that over half of these graphics were created toward the end of the 20th century, when the sole basis for their creation was field observations. This was a period when systematic botany lacked widely available computer tools for data acquisition, collection, processing, and presentation [61,62,63,64]. Secondly, and more importantly, *E*. *helleborine* is a highly morphologically variable species, which may pose a challenge to the generalisation of its structural pattern [6,13,14,17,18,53,65,66,67,68,69,70,71]. The observed freedom of European researchers in graphically interpreting the morphology of the *E*. *helleborine* gynostemium raises doubts about the significance of the structural pattern of this floral organ in the identification of other closely related species of the genus *Epipactis*. The taxonomic value of its form is also questioned.

Despite the considerable popularity of geometric morphometric methods (GMMs) in systematic botany, no studies have yet been conducted on such a complex floral organ as the orchid gynostemium. The only published study that could provide new insights into the use of morphometric parameters of the gynostemium in the taxonomy of *E*. *helleborine* is the study by Urbanek Krajnc et al. [72]. Despite the obvious genetic differences observed between the taxa studied, the researchers noted a considerable range of variability in the characteristics of the gynostemium structures. This makes it difficult to distinguish between individuals of the *E*. *helleborine* agg. and the *E*. *leptochila* agg. Unfortunately, the research material used for comparison is limited in quantity and origin. This may raise questions about the usefulness of the results obtained.

In light of our findings, the paucity of published morphometric data is also noteworthy, particularly concerning the length, width, and height of the gynostemium observed in the species studied. The wider availability of such data would allow us to answer the question of whether these parameters are characteristic of specific species and, more importantly, whether taxonomic identification can be effectively carried out on the basis of these parameters alone. A comparison of the mean values of the metric parameters obtained during the study for the gynostemium in *E*. *helleborine* with similar data obtained for *E*. *purpurata* [57] suggests that the reported characteristics differ sufficiently to allow the two species to be distinguished on the basis of them. It should be noted that these species can also be distinguished by other, more easily verifiable characteristics, such as the purple shoot or perianth characteristic of *E*. *purpurata*. However, in cases where herbarium specimens are damaged or poorly preserved, or when analysing the colour forms of both species, such characteristics may prove inconclusive. It is not always possible to obtain samples of material for definitive genetic analysis.

An undoubtedly important result of the unpublished research was the finding that the taxonomic identification of *E*. *helleborine* based solely on the morphological structural pattern of the gynostemium dissected from the flower bud is not justified [34]. This is mainly due to the high heterogeneity of the structural pattern of the gynostemium in the juvenile stage of development. This is particularly significant considering that herbarium sheets containing plants with flowers in the bud stage or ramets just beginning to flower constitute a large part of herbarium collections, and in the past, were sometimes even preserved as holotypes [73]. The results demonstrate that only gynostemia of fully developed flowers show relative stability in their structural pattern. This finding supports the view that, in the case of *Epipactis* species, only fully flowering ramets are suitable for identification and especially for documenting new taxa [74].

Within the taxonomic classification of *E*. *helleborine*, is the morphology of the gynostemium structure a useful diagnostic characteristic? Unfortunately, the results obtained are not entirely clear. None of the morphometric parameters examined allowed the differentiation of specific populations. This supports the conclusion that all the ramets in the study are members of the same species. Consequently, the observed morphological variability of the gynostemium cannot be used as a basis for determining infraspecific taxa. The distinctiveness of all populations is evident, as demonstrated by the consistent and distinctive structural pattern of the gynostemium produced each year. However, it was not possible to identify the population from which it originated based on a single morphometric parameter.

The distinctive structural pattern of the gynostemium may be attributed to a combination of microevolutionary processes, including dominant vegetative multiplication, population isolation, and limited gene flow. Additionally, the species’ relatively young age might also contribute. This is, of course, a convenient explanation, arising from the lack of sufficient data to rationally explain the observed phenomenon. Nevertheless, the capacity of each isolated population to produce a distinctive gynostemium-shaped pattern annually suggests that researchers should exercise caution when making taxonomic identification.

This raises the question of whether it would be justified to distinguish and describe new species based solely on the structural pattern of the gynostemium. Some insight may be provided by recent genetic studies of the genus *Epipactis*, which indicate weak divergence of the group and suggest that the separation of aggregate taxa, rather than local and controversial species, may be a more valid approach [75,76].

## 5. Conclusions

The morphological variability of the gynostemium in *E*. *helleborine* is characterised by continuous variation in characteristics such as length, width, height, and the angle of inclination of the stigma. This variability is more pronounced between populations than within populations. This suggests that population-level differences have a significant influence on the morphological characteristics of the gynostemium. The observed differences in gynostemium shape, driven by nominal variables such as population, ramet, and survey year, indicate the high level of natural variability of this floral organ. This variability may limit the reliability of using gynostemium shape as the sole diagnostic characteristic for taxonomic identification. However, within individual ramets, the morphological characteristics of the gynostemium—particularly its length, width, height, and angle of stigma inclination—are sufficiently consistent to support accurate species identification based on each fully developed gynostemium.

Our findings highlight the crucial role of population-level variation in shaping the morphological characteristics of the gynostemium. Although intra-population variation can still be informative, it is primarily the inter-population differences that provide the strongest diagnostic potential. Therefore, previous reliance solely on a single view of the gynostemium—without the support of additional morphological characteristics visible from other views—may lead to misidentification.

As a result, the development of gynostemium morphometrics, including size and angle of stigma inclination, combined with graphical representations of its morphological structural pattern, would increase the accuracy of species identification, thereby improving taxonomic clarity and reliability of classification within the genus *Epipactis*.

Therefore, we recommend that future taxonomic keys for the *Epipactis* species include detailed illustrations of the general structural pattern of the gynostemium, showing its morphology from each of the four standard viewing directions: dorsal, frontal, ventral, and right lateral.

It should be noted that, due to the methodology used, the research focuses on the regional differences of *E*. *helleborine* in southern Poland, which limits the ability to make broader generalisations. Therefore, future studies should aim to include a significantly larger number of populations, thus providing a more comprehensive understanding of the natural range of the species.

## Figures and Tables

**Table 1:** Geographic location, sample size, and phytosociological characteristics of the studied populations.

Population	Geographic Location		Number of Collected Flowers	Phytosociological Characteristics *
	Latitude (N)	Longitude (E)		
Kotowice	50°1′59′′	17°12′54′′	23	Riparian forest *Ficario-Ulmetum minoris* Knapp 1942 em. J. Mat. 1976
Milicz	51°31′5′′	17°15′40′′	27	Anthropogenically modified forests *Quercetea robori-petraeae* Br.-Bl. Et R. Tx. 1943
Trestno	51°4′47′′	17°8′5′′	37	Regenerative community of riparian shrub-forest *Salicitea purpureae* Moor 1958
Żelazno	50°21′22′′	16°40′59′′	35	Degenerated form of orchid beech forests *Cephalanthero-Fagenion* Tüxen et Oberdorfer 1958

* According to [36].

## Data Availability

The raw data are attached in the Supplementary Materials.

## Supplementary Materials

The following supporting information can be downloaded at: www.mdpi.com/article/10.3390/biology14030241/s1, Table S1: Size and angle of stigma inclination measurements of the gynostemium in *Epipactis helleborine*; Table S2: The results of Procrustes ANOVA and MANOVA (Pillai’s trace) of gynostemium shape variation in *Epipactis helleborine* among studied populations, ramets, and years of research; Table S3: The results of global tests for the determination of significant differences among the means of studied populations, ramets, and years of research; Table S4: Mahalanobis distances computed from canonical variate analysis (CVA) of the covariance matrices generated on averaged data for 122 gynostemia in *Epipactis helleborine* from different populations; Table S5: Procrustes distances computed from canonical variate analysis (CVA) of the covariance matrices generated on averaged data for 122 gynostemia in *Epipactis helleborine* from different populations; Table S6: Mahalanobis distances computed from canonical variate analysis (CVA) of the covariance matrices generated on averaged data for 122 gynostemia in *Epipactis helleborine* from different ramets; Table S7: Procrustes distances computed from canonical variate analysis (CVA) of the covariance matrices generated on averaged data for 122 gynostemia in *Epipactis helleborine* from different ramets; Table S8: Mahalanobis distances computed from canonical variate analysis (CVA) of the covariance matrices generated on averaged data for 122 gynostemia in *Epipactis helleborine* from different years of research; Table S9: Procrustes distances computed from canonical variate analysis (CVA) of the covariance matrices generated on averaged data for 122 gynostemia in *Epipactis helleborine* from different years of research; Table S10: Descriptive statistics of all morphometric parameters of the gynostemium in *Epipactis helleborine* from different populations; Table S11: Descriptive statistics of all morphometric parameters of the gynostemium in *Epipactis helleborine* from different ramets; Table S12: Descriptive statistics of all morphometric parameters of the gynostemium in *Epipactis helleborine* from different years of research; Figure S1. Plot of the first two principal components (PCs) from principal component analysis for 122 gynostemium configurations in dorsal view; Figure S2: Plot of the first two principal components (PCs) from principal component analysis for 122 gynostemium configurations in frontal view; Figure S3: Plot of the first two principal components (PCs) from principal component analysis for 122 gynostemium configurations in ventral view; Figure S4: Plot of the first two principal components (PCs) from principal component analysis for 122 gynostemium configurations in right lateral view; Figure S5: Wireframe graphs describing gynostemium shape changes between the minimum and maximum values of PC1 and PC2; Figure S6: Plot of the first two canonical variates (CVs) from canonical variate analysis conducted on averaged data for 122 gynostemia in dorsal view; Figure S7: Plot of the first two canonical variates (CVs) from canonical variate analysis conducted on averaged data for 122 gynostemia in frontal view; Figure S8: Plot of the first two canonical variates (CVs) from canonical variate analysis conducted on averaged data for 122 gynostemia in ventral view; Figure S9: Plot of the first two canonical variates (CVs) from canonical variate analysis conducted on averaged data for 122 gynostemia in right lateral view; Figure S10: Wireframe graphs describing gynostemium shape changes in dorsal view between the minimum and maximum values of CV1 and CV2; Figure S11: Wireframe graphs describing gynostemium shape changes in frontal view between the minimum and maximum values of CV1 and CV2; Figure S12: Wireframe graphs describing gynostemium shape changes in ventral view between the minimum and maximum values of CV1 and CV2; Figure S13. Wireframe graphs describing gynostemium shape changes in right lateral view between the minimum and maximum values of CV1 and CV2.
