# Supplementary material for: Shape and Size Variability of the Gynostemium in Epipactis helleborine (L.) Crantz (Orchidaceae)
Source: Biology (Basel). 2025 Feb 27;14(3):241. doi: 10.3390/biology14030241 (PMC11939435; doi:10.3390/biology14030241)
Supplement: Supplementary file 1 [file biology-14-00241-s001.zip › biology-3474812-supplementary.pdf]

Table S1. Size and angle of stigma inclination measurements of the gynostemium in *Epipactis helleborine*.

No.	Year of collection	Population	Number of the ramet	Length [mm]	Width [mm]	Height [mm]	Stigma inclination angle [°]
1	2017	Kotowice	1	4.90	2.88	2.49	62.40
2	2017	Kotowice	1	5.20	2.78	2.40	78.26
3	2017	Kotowice	1	5.01	2.81	2.82	84.22
4	2017	Kotowice	1	5.39	3.17	2.75	61.78
5	2017	Kotowice	1	5.03	2.96	2.83	82.46
6	2017	Kotowice	1	5.07	2.95	2.90	74.36
7	2017	Kotowice	1	4.71	2.58	2.57	95.32
8	2017	Kotowice	1	5.03	2.80	2.64	78.43
9	2018	Kotowice	2	5.16	3.06	3.03	57.66
10	2018	Kotowice	2	4.79	2.79	2.63	63.24
11	2018	Kotowice	2	5.04	2.83	2.76	63.59
12	2018	Kotowice	2	5.26	3.15	3.17	53.93
13	2018	Kotowice	2	5.06	2.55	2.53	72.42
14	2018	Kotowice	2	5.24	2.62	2.58	68.49
15	2019	Kotowice	3	4.95	3.29	3.63	53.99
16	2019	Kotowice	3	5.63	3.35	3.73	56.31
17	2019	Kotowice	3	4.79	2.86	3.06	63.69
18	2019	Kotowice	3	5.35	2.98	3.31	59.94
19	2019	Kotowice	3	5.02	2.81	3.21	45.21
20	2019	Kotowice	3	4.72	2.88	2.96	51.52
21	2019	Kotowice	3	4.99	2.74	3.33	49.82
22	2019	Kotowice	3	4.62	2.50	2.67	53.12
23	2019	Kotowice	3	4.81	2.62	3.05	52.36
24	2017	Milicz	4	4.43	2.68	2.64	64.15
25	2017	Milicz	4	4.49	2.66	2.47	64.42
26	2017	Milicz	4	4.40	2.68	2.55	65.97
27	2017	Milicz	4	4.42	2.60	2.65	60.05
28	2017	Milicz	4	4.16	2.47	2.34	73.08
29	2017	Milicz	4	4.37	2.54	2.55	70.91
30	2017	Milicz	4	4.22	2.22	2.24	77.01
31	2017	Milicz	4	3.90	2.23	2.19	78.51
32	2018	Milicz	5	4.47	2.75	2.58	56.31
33	2018	Milicz	5	4.34	2.71	2.52	73.83
34	2018	Milicz	5	4.48	2.86	2.84	65.88
35	2018	Milicz	5	3.96	2.53	2.21	81.30
36	2018	Milicz	5	4.17	2.53	2.27	81.37
37	2018	Milicz	5	4.24	2.60	2.26	87.46
38	2019	Milicz	6	4.56	2.62	2.52	83.38
39	2019	Milicz	6	4.92	2.83	2.94	69.74
40	2019	Milicz	6	4.89	2.83	2.87	65.41
41	2019	Milicz	6	4.89	2.79	2.82	63.86
42	2019	Milicz	6	4.93	2.81	2.94	74.51
43	2019	Milicz	6	4.46	2.91	2.67	67.63
44	2019	Milicz	6	4.82	2.87	2.55	74.85
45	2019	Milicz	6	4.34	2.85	2.86	74.97
46	2019	Milicz	6	4.21	2.71	2.30	69.11
47	2019	Milicz	6	4.77	2.43	2.33	77.29
48	2019	Milicz	6	4.43	2.80	2.61	76.69
49	2019	Milicz	6	4.58	2.65	2.75	70.12
50	2019	Milicz	6	4.33	2.52	2.58	76.44
51	2017	Trestno	7	4.27	2.76	3.19	47.22
52	2017	Trestno	7	4.82	2.81	3.34	40.64
53	2017	Trestno	7	4.62	3.07	3.40	41.51
54	2017	Trestno	7	4.92	2.81	3.12	47.44
55	2017	Trestno	7	4.53	3.02	3.38	47.23
56	2017	Trestno	7	4.37	2.51	2.58	43.48
57	2017	Trestno	7	4.68	2.70	2.68	51.35
58	2017	Trestno	7	4.45	2.52	2.66	42.61
59	2017	Trestno	7	4.73	2.79	2.82	48.20
60	2017	Trestno	7	4.20	2.56	2.68	45.11
61	2017	Trestno	7	4.17	2.59	2.80	60.49
62	2018	Trestno	8	4.35	2.62	2.85	36.93
63	2018	Trestno	8	4.27	3.12	3.63	45.26
64	2018	Trestno	8	4.54	2.77	3.19	46.22
65	2018	Trestno	8	4.87	2.93	3.56	51.77
66	2018	Trestno	8	4.78	2.87	3.38	45.53
67	2018	Trestno	8	4.42	2.69	3.30	46.76
68	2018	Trestno	8	4.46	2.70	3.26	39.46
69	2018	Trestno	8	4.44	2.38	2.74	47.83
70	2018	Trestno	8	4.22	2.96	3.73	41.97
71	2018	Trestno	8	4.05	2.97	3.49	43.57
72	2018	Trestno	8	4.89	2.60	3.17	51.75
73	2018	Trestno	8	4.81	2.60	3.08	55.04
74	2018	Trestno	8	4.90	2.66	3.15	55.13
75	2018	Trestno	8	4.85	2.65	3.31	40.21
76	2018	Trestno	8	4.82	2.58	3.01	51.58
77	2018	Trestno	8	4.45	2.62	3.17	54.49
78	2019	Trestno	9	5.38	3.28	3.83	46.95
79	2019	Trestno	9	5.23	3.29	3.56	43.78
80	2019	Trestno	9	5.48	3.39	3.46	51.23

81	2019	Trestno	9	5.12	3.15	3.34	52.38
82	2019	Trestno	9	5.04	3.27	3.57	47.02
83	2019	Trestno	9	4.82	3.26	3.61	46.78
84	2019	Trestno	9	4.64	3.05	3.40	42.68
85	2019	Trestno	9	4.59	3.15	3.48	44.80
86	2019	Trestno	9	4.80	3.03	3.44	51.31
87	2019	Trestno	9	4.86	2.99	3.47	59.67
88	2017	Żelazno	10	4.31	3.13	2.76	59.14
89	2017	Żelazno	10	4.64	2.96	2.53	59.92
90	2017	Żelazno	10	3.95	2.89	2.81	68.64
91	2017	Żelazno	10	3.76	2.98	2.57	63.59
92	2017	Żelazno	10	4.13	2.81	2.69	60.90
93	2017	Żelazno	10	4.11	2.66	2.65	67.01
94	2017	Żelazno	10	4.31	2.69	2.63	63.26
95	2017	Żelazno	10	4.05	2.71	2.78	63.44
96	2017	Żelazno	10	4.34	2.40	2.63	74.08
97	2018	Żelazno	11	4.80	3.38	3.04	70.50
98	2018	Żelazno	11	4.68	3.31	3.12	70.40
99	2018	Żelazno	11	4.33	3.31	2.95	68.20
100	2018	Żelazno	11	4.54	3.29	3.09	73.96
101	2018	Żelazno	11	4.49	3.15	2.63	78.92
102	2018	Żelazno	11	4.83	3.18	3.00	74.44
103	2018	Żelazno	11	5.11	3.13	2.90	77.42
104	2018	Żelazno	11	5.07	3.08	2.79	77.13
105	2018	Żelazno	11	4.83	3.29	3.03	71.43
106	2018	Żelazno	11	4.72	3.14	2.88	78.28
107	2018	Żelazno	11	4.70	3.06	2.96	78.71
108	2018	Żelazno	11	4.73	3.06	2.95	78.78
109	2018	Żelazno	11	4.91	2.98	2.70	68.92
110	2018	Żelazno	11	4.62	2.99	2.88	81.68
111	2018	Żelazno	11	4.71	2.99	2.65	81.46
112	2018	Żelazno	11	4.71	2.79	2.60	74.90
113	2019	Żelazno	12	5.47	3.44	3.65	63.36
114	2019	Żelazno	12	5.26	3.64	3.79	53.40
115	2019	Żelazno	12	5.28	3.48	3.71	63.75
116	2019	Żelazno	12	5.26	3.50	3.68	60.41
117	2019	Żelazno	12	5.01	3.29	3.49	56.28
118	2019	Żelazno	12	5.14	3.16	3.23	67.86
119	2019	Żelazno	12	4.65	3.29	3.53	68.32
120	2019	Żelazno	12	4.39	3.15	3.45	68.16
121	2019	Żelazno	12	4.76	3.02	3.31	68.10
122	2019	Żelazno	12	4.80	3.02	3.42	70.10

Table S2. The results of Procrustes ANOVA and MANOVA (Pillai's trace) of gynostemium shape variation in *Epipactis helleborine* among studied populations, ramets, and years of research.

View	Effect	MS	df	F	*p*-value	Pillai's trace	*p*-value
dorsal	population	0.0076162204	57	36.07	< 0.0001	2.22	< 0.0001
	ramet	0.0048491506	209	24.35	< 0.0001	5.88	< 0.0001
	year of research	0.0037538788	38	31.94	< 0.0001	1.05	< 0.0001
frontal	population	0.0090800375	87	27.02	< 0.0001	2.22	< 0.0001
	ramet	0.0058723747	319	30.90	< 0.0001	7.75	< 0.0001
	year of research	0.0072746002	58	52.21	< 0.0001	1.44	< 0.0001
ventral	population	0.0045679494	96	46.19	< 0.0001	2.56	< 0.0001
	ramet	0.0034414255	352	33.43	< 0.0001	8.59	< 0.0001
	year of research	0.0035940851	64	37.63	< 0.0001	1.57	< 0.0001
right lateral	population	0.0101415664	72	17.87	< 0.0001	2.13	< 0.0001
	ramet	0.0051343232	264	13.81	< 0.0001	6.67	< 0.0001
	year of research	0.0023090692	48	2.96	< 0.0001	1.08	< 0.0001

Table S3. The results of global tests for the determination of significant differences among the means of studied populations, ramets, and years of research.

View	Effect	Goodall's F	*p*-value	Pillai's trace	*p*-value
	population	12.5477	< 0.0001	2.2235	< 0.0001
dorsal	ramet	12.9683	< 0.0001	5.8843	< 0.0001
	year of research	5.1367	< 0.0001	1.0466	< 0.0001
	population	13.8245	< 0.0001	2.2237	< 0.0001
frontal	ramet	16.0898	< 0.0001	7.7522	< 0.0001
	year of research	9.5979	< 0.0001	1.4360	< 0.0001
	population	12.2751	< 0.0001	2.5578	< 0.0001
ventral	ramet	19.1579	< 0.0001	8.5915	< 0.0001
	year of research	8.4814	< 0.0001	1.5713	< 0.0001
	population	17.8734	< 0.0001	2.1290	< 0.0001
right lateral	ramet	13.8081	< 0.0001	6.6743	< 0.0001
	year of research	2.9622	0.0011*	1.0849	< 0.0001

Table S4. Mahalanobis distances computed from canonical variate analysis (CVA) of the covariance matrices generated on averaged data for 122 gynostemia in *Epipactis helleborine* from different populations.

Dorsal	Kotowice	Milicz	Trestno	Żelazno
**Kotowice**	–	< 0.0001	< 0.0001	< 0.0001
**Milicz**	5.7014	–	< 0.0001	< 0.0001
**Trestno**	4.3554	4.5563	–	< 0.0001
**Żelazno**	4.8020	5.2510	4.8393	–
**Frontal**	**Kotowice**	**Milicz**	**Trestno**	**Żelazno**
**Kotowice**	–	< 0.0001	< 0.0001	< 0.0001
**Milicz**	4.3090	–	< 0.0001	< 0.0001
**Trestno**	7.0472	7.8000	–	< 0.0001
**Żelazno**	3.6464	5.9934	7.6524	–
**Ventral**	**Kotowice**	**Milicz**	**Trestno**	**Żelazno**
**Kotowice**	–	< 0.0001	< 0.0001	< 0.0001
**Milicz**	9.5819	–	< 0.0001	< 0.0001
**Trestno**	5.6601	8.0773	–	< 0.0001
**Żelazno**	6.8004	8.3297	7.5430	–
**Right lateral**	**Kotowice**	**Milicz**	**Trestno**	**Żelazno**
**Kotowice**	–	< 0.0001	< 0.0001	< 0.0001
**Milicz**	3.8898	–	< 0.0001	< 0.0001
**Trestno**	4.8881	5.9975	–	< 0.0001
**Żelazno**	4.8023	4.0549	5.5056	–

Table S5. Procrustes distances computed from canonical variate analysis (CVA) of the covariance matrices generated on averaged data for 122 gynostemia in *Epipactis helleborine* from different populations.

**Dorsal**	**Kotowice**	**Milicz**	**Trestno**	**Żelazno**
**Kotowice**	–	0.0049*	< 0.0001	< 0.0001
**Milicz**	0.0621	–	< 0.0001	0.0001*
**Trestno**	0.1212	0.1185	–	< 0.0001
**Żelazno**	0.0789	0.0791	0.1007	–
**Frontal**	**Kotowice**	**Milicz**	**Trestno**	**Żelazno**
**Kotowice**	–	0.0333*	< 0.0001	0.0156*
**Milicz**	0.0718	–	< 0.0001	0.0105*
**Trestno**	0.1777	0.1535	–	< 0.0001
**Żelazno**	0.0668	0.0684	0.1638	–
**Ventral**	**Kotowice**	**Milicz**	**Trestno**	**Żelazno**
**Kotowice**	–	< 0.0001	< 0.0001	0.0007*
**Milicz**	0.0854	–	< 0.0001	< 0.0001
**Trestno**	0.1015	0.1059	–	< 0.0001
**Żelazno**	0.0694	0.1009	0.1095	–
**Right lateral**	**Kotowice**	**Milicz**	**Trestno**	**Żelazno**
**Kotowice**	–	< 0.0001	< 0.0001	< 0.0001
**Milicz**	0.0935	–	< 0.0001	< 0.0001
**Trestno**	0.1297	0.1607	–	< 0.0001
**Żelazno**	0.0916	0.0756	0.1557	–

* statistically non-significant result

Table S6. Mahalanobis distances computed from canonical variate analysis (CVA) of the covariance matrices generated on averaged data for 122 gynostemia in *Epipactis helleborine* from different ramets.

**Dorsal**	**1**	**2**	**3**	**4**	**5**	**6**	**7**	**8**	**9**	**10**	**11**	**12**
**1**	–	0.2121*	0.7261*	0.3922*	0.2879*	0.1494*	0.6938*	0.0002*	0.3433*	0.9839*	0.0133*	0.0818*
**2**	7.7129	–	0.8730*	0.0611*	0.6732*	0.5332*	0.9964*	0.2315*	0.0988*	0.7711*	0.0110*	0.9334*
**3**	4.7328	6.6258	–	0.0109*	0.2845*	0.1272*	0.0012*	0.0005*	1.0000*	0.9981*	0.0153*	0.1571*
**4**	7.9788	10.8730	6.4666	–	0.4564*	0.3397*	1.0000*	0.0044*	0.3644*	0.6926*	0.0003*	0.4289*
**5**	9.6873	10.7589	8.5931	6.2429	–	0.0016*	0.0072*	0.0678*	0.6781*	0.3137*	0.0018*	0.0101*
**6**	7.7007	7.7840	6.6764	6.8365	5.4805	–	0.0069*	< 0.0001	0.0309*	0.0744*	0.0002*	0.0002*
**7**	6.9553	6.6048	6.0623	8.9276	9.0421	6.6374	–	0.0006*	0.1135*	0.9989*	< 0.0001	0.0437*
**8**	9.2139	6.5088	7.7486	8.6543	7.9909	6.1850	7.8738	–	0.0197*	< 0.0001	< 0.0001	< 0.0001
**9**	7.8944	4.5034	6.6074	9.3413	8.6465	5.4829	4.3166	5.6456	–	0.8083*	0.0013*	0.0001*
**10**	6.9377	9.8221	8.7298	9.1350	9.5717	7.6165	9.4687	8.3993	8.8350	–	0.0073*	0.0003*
**11**	7.0343	6.2392	6.9074	8.6102	9.5093	6.7795	8.0428	6.3160	6.2067	6.2526	–	0.0005*
**12**	7.0905	10.1796	6.5876	5.4001	6.7664	7.8348	8.2078	8.6292	8.9825	7.8142	7.4679	–
**Frontal**	**1**	**2**	**3**	**4**	**5**	**6**	**7**	**8**	**9**	**10**	**11**	**12**
**1**	–	0.1104*	0.0091*	0.0793*	0.3886*	0.9295*	0.4496*	0.0170*	0.1380*	0.0418*	0.7922*	0.0079*
**2**	9.1147	–	0.7582*	0.3299*	0.0938*	0.0435*	0.0030*	0.4836*	0.9955*	0.0185*	0.5120*	0.5114*
**3**	10.0633	9.2061	–	0.1107*	0.2100*	0.5103*	0.4300*	0.4418*	0.9379*	0.1550*	0.0644*	0.1774*
**4**	10.4585	12.8120	10.1156	–	0.2713*	0.6776*	0.5467*	0.9999*	0.9138*	0.2942*	0.8459*	0.8818*
**5**	12.6190	11.5598	9.7425	13.6465	–	0.3791*	0.0615*	0.7714*	0.0048*	0.2287*	0.9744*	0.1029*
**6**	8.8955	8.7465	8.7304	12.9428	10.6616	–	0.3693*	0.0005*	0.0083*	0.1950*	0.9850*	0.0264*
**7**	10.5597	9.3191	8.1458	10.6538	12.2264	9.4860	–	0.7068*	0.4943*	0.5521*	1.0000*	0.7285*
**8**	14.1406	12.9653	11.2484	13.9325	16.5577	12.6770	7.8303	–	0.6138*	0.7595*	0.0116*	0.2313*
**9**	13.8759	11.4148	10.1083	14.0836	15.0181	11.1234	7.4301	5.5667	–	0.0060*	0.0001*	0.3474*
**10**	10.9190	11.6295	11.1250	12.1642	12.0212	12.9912	11.5265	16.8875	16.9261	–	0.1351*	0.9536*
**11**	6.8390	6.6103	8.0592	11.8368	10.4486	9.3099	9.8486	14.5001	13.1066	9.3035	–	0.9972*
**12**	10.4523	10.2463	7.6892	8.8337	11.1542	10.7433	7.9890	12.2007	11.0922	10.4488	9.2477	–
**Ventral**	**1**	**2**	**3**	**4**	**5**	**6**	**7**	**8**	**9**	**10**	**11**	**12**
**1**	–	0.5579*	0.5677*	0.2543*	0.4189*	0.4697*	0.3899*	0.0158*	0.9987*	0.6321*	0.1541*	0.6414*
**2**	11.2695	–	0.1983*	0.1311*	0.5583*	0.2237*	0.2851*	0.2679*	0.0486*	0.7777*	0.4400*	0.8484*
**3**	11.4549	7.4850	–	0.9971*	0.0861*	0.3003*	0.1182*	0.0545*	0.3428*	0.6677*	0.5908*	0.4521*
**4**	17.0772	14.5633	14.0466	–	0.6868*	0.9837*	0.7976*	0.9884*	0.9288*	0.9353*	0.9858*	0.9999*
**5**	17.7013	13.3239	14.0675	12.8712	–	0.0341*	0.1489*	0.0602*	0.5941*	0.5524*	0.9349*	0.4201*
**6**	12.5700	13.0454	10.5125	13.8175	12.3535	–	0.9988*	0.1498*	0.8392*	0.8217*	0.4920*	0.2403*
**7**	11.6359	8.9506	9.8932	14.2710	12.1393	11.2014	–	0.0619*	0.2128*	0.9088*	< 0.0001	0.6711*
**8**	13.3006	9.4443	6.9267	11.6348	12.7776	11.5708	10.4258	–	0.0211*	0.1255*	0.0022*	0.0572*
**9**	14.8635	10.8515	12.2279	13.2912	13.2366	13.5568	8.7182	11.7854	–	0.0409*	0.0300*	0.6538*
**10**	17.0105	15.3543	15.5507	17.7201	18.9852	17.8663	16.9200	16.5772	18.1274	–	0.9505*	0.6813*
**11**	13.9360	12.2454	11.6624	17.4695	14.6566	12.8287	13.3527	14.2713	14.1143	14.1527	–	0.9707*
**12**	11.7280	9.3473	9.8508	12.7189	9.8717	11.1740	9.9553	9.7990	10.1681	14.2981	11.2668	–
**Right lateral**	**1**	**2**	**3**	**4**	**5**	**6**	**7**	**8**	**9**	**10**	**11**	**12**
**1**	–	0.0921*	0.2280*	0.0399*	0.9703*	0.8924*	0.9631*	0.9188*	0.3680*	0.5016*	0.1010*	0.4472*
**2**	8.9383	–	0.6942*	0.1690*	0.7297*	0.4177*	0.5089*	0.9767*	0.3274*	0.9872*	0.0635*	0.0060*
**3**	6.4430	7.9851	–	0.2989*	0.1487*	0.9991*	0.6703*	0.9853*	0.2915*	0.4145*	0.0012*	0.9122*
**4**	8.4224	8.3118	7.1923	–	0.0502*	0.7528*	0.9895*	0.6492*	0.0759*	0.0002*	0.0001*	0.5701*
**5**	10.9078	9.8745	11.4530	9.4192	–	0.7840*	0.0709*	0.0046*	0.9057*	0.8909*	1.0000*	0.6507*
**6**	7.4259	6.0268	6.7177	6.8077	8.1768	–	0.6596*	0.0064*	0.9811*	0.6639*	0.0077*	0.9998*
**7**	9.3515	10.1838	5.0414	7.6470	12.0863	8.5344	–	0.0160*	0.6062*	< 0.0001	0.0018*	0.9973*
**8**	8.7988	10.2589	5.0975	8.1123	12.6879	8.7839	4.9389	–	0.0860*	0.0089*	0.0001*	0.0563*
**9**	8.4290	9.7828	5.5303	8.0624	12.3269	7.6622	4.2928	4.6594	–	0.6266*	0.0260*	0.4942*
**10**	9.4424	11.1748	9.2974	8.2040	9.5405	9.5641	9.6676	9.9888	9.9666	–	0.9996*	0.3578*
**11**	6.0063	8.8652	6.6854	7.8951	11.6459	7.8666	8.5257	8.5302	8.0495	7.5726	–	0.1313*
**12**	10.5357	9.9561	9.2389	5.1454	11.0345	7.7771	8.2962	9.3731	7.8176	10.0252	9.9635	–

* statistically non-significant result

Table S7. Procrustes distances computed from canonical variate analysis (CVA) of the covariance matrices generated on averaged data for 122 gynostemia in *Epipactis helleborine* from different ramets.

**Dorsal**	**1**	**2**	**3**	**4**	**5**	**6**	**7**	**8**	**9**	**10**	**11**	**12**
**1**	–	0.0003*	0.0002*	0.0115*	< 0.0001	< 0.0001	< 0.0001	< 0.0001	< 0.0001	0.0102*	< 0.0001	0.0003*
**2**	0.1639	–	0.0003*	0.0005*	0.0019*	< 0.0001	0.0001*	0.0004*	0.0060*	0.0021*	0.0003*	0.0001*
**3**	0.0859	0.1122	–	0.0012*	< 0.0001	< 0.0001	< 0.0001	< 0.0001	< 0.0001	0.0001*	< 0.0001	< 0.0001
**4**	0.0905	0.1992	0.1055	–	0.0086*	< 0.0001	< 0.0001	< 0.0001	0.0001*	0.0015*	< 0.0001	0.0008*
**5**	0.0974	0.1641	0.1098	0.1051	–	< 0.0001	< 0.0001	< 0.0001	0.0002*	0.0029*	< 0.0001	0.0026*
**6**	0.1306	0.0867	0.1043	0.1657	0.1043	–	< 0.0001	< 0.0001	< 0.0001	0.0007*	< 0.0001	< 0.0001
**7**	0.2011	0.1226	0.1693	0.2331	0.1834	0.1184	–	< 0.0001	0.0002*	< 0.0001	< 0.0001	< 0.0001
**8**	0.1672	0.0942	0.1382	0.1939	0.1303	0.0667	0.1249	–	0.0001*	0.0001*		
**9**	0.1943	0.0695	0.1431	0.2250	0.1801	0.0959	0.0820	0.1016	–	< 0.0001	< 0.0001	< 0.0001
**10**	0.0933	0.1382	0.1068	0.1355	0.0971	0.0958	0.1664	0.1218	0.1528	–	< 0.0001	0.0012*
**11**	0.1628	0.0869	0.1196	0.1926	0.1638	0.1054	0.1332	0.1285	0.0895	0.1202	–	< 0.0001
**12**	0.1136	0.1699	0.1208	0.1242	0.0885	0.1203	0.1537	0.1474	0.1668	0.0982	0.1409	–
**Frontal**	**1**	**2**	**3**	**4**	**5**	**6**	**7**	**8**	**9**	**10**	**11**	**12**
**1**	–	0,0005*	< 0.0001	0,0008*	0,0005*	< 0.0001	0,0001*	< 0.0001	< 0.0001	< 0.0001	0,0005*	< 0.0001
**2**	0,1425	–	0,0001*	< 0.0001	0,0003*	< 0.0001	< 0.0001	< 0.0001	< 0.0001	0,0002*	0,0153*	0,0001*
**3**	0,2090	0,1507	–	< 0.0001	< 0.0001	< 0.0001	< 0.0001	< 0.0001	< 0.0001	< 0.0001	< 0.0001	< 0.0001
**4**	0,1300	0,1978	0,2247	–	0,0001*	< 0.0001	< 0.0001	< 0.0001	0,0001*	< 0.0001	< 0.0001	< 0.0001
**5**	0,1867	0,1664	0,1574	0,1938	–	< 0.0001	< 0.0001	< 0.0001	< 0.0001	< 0.0001	< 0.0001	0,0153*
**6**	0,2151	0,1548	0,1008	0,2551	0,1598	–	< 0.0001	< 0.0001	< 0.0001	< 0.0001	< 0.0001	< 0.0001
**7**	0,2538	0,1953	0,1115	0,2446	0,1687	0,1406	–	< 0.0001	< 0.0001	< 0.0001	< 0.0001	< 0.0001
**8**	0,2740	0,2256	0,1308	0,2788	0,1954	0,1431	0,1036	–	0,0008*	< 0.0001	< 0.0001	< 0.0001
**9**	0,2926	0,2240	0,1369	0,2969	0,2111	0,1597	0,0947	0,0814	–	< 0.0001	< 0.0001	< 0.0001
**10**	0,1663	0,1672	0,1678	0,1937	0,1806	0,1697	0,2020	0,2473	0,2690	–	< 0.0001	< 0.0001
**11**	0,1258	0,0895	0,1311	0,1709	0,1560	0,1631	0,1828	0,2230	0,2205	0,1471	–	< 0.0001
**12**	0,2447	0,2107	0,1225	0,2211	0,1517	0,1500	0,0943	0,1312	0,1436	0,1932	0,1919	–
**Ventral**	**1**	**2**	**3**	**4**	**5**	**6**	**7**	**8**	**9**	**10**	**11**	**12**
**1**	–	< 0.0001	< 0.0001	0,0002*	< 0.0001	< 0.0001	< 0.0001	< 0.0001	< 0.0001	< 0.0001	< 0.0001	< 0.0001
**2**	0,1367	–	0,0001*	0,0003*	0,0015*	< 0.0001	< 0.0001	< 0.0001	< 0.0001	0,0003*	< 0.0001	< 0.0001
**3**	0,1226	0,0668	–	< 0.0001	< 0.0001	< 0.0001	< 0.0001	< 0.0001	< 0.0001	< 0.0001	< 0.0001	< 0.0001
**4**	0,1505	0,1937	0,1813	–	0,0005*	< 0.0001	< 0.0001	< 0.0001	< 0.0001	< 0.0001	< 0.0001	< 0.0001
**5**	0,1370	0,1418	0,1298	0,1225	–	< 0.0001	0,0004*	< 0.0001	< 0.0001	< 0.0001	< 0.0001	0,0015*
**6**	0,1185	0,0898	0,0719	0,1626	0,1071	–	< 0.0001	< 0.0001	< 0.0001	< 0.0001	< 0.0001	< 0.0001
**7**	0,1483	0,1237	0,1192	0,1831	0,1191	0,1039	–	< 0.0001	< 0.0001	< 0.0001	< 0.0001	< 0.0001
**8**	0,1438	0,1210	0,0907	0,1741	0,1173	0,0757	0,0922	–	< 0.0001	< 0.0001	< 0.0001	< 0.0001
**9**	0,1829	0,1605	0,1547	0,2369	0,1875	0,1527	0,1027	0,1413	–	< 0.0001	< 0.0001	< 0.0001
**10**	0,1837	0,1735	0,1765	0,1877	0,1745	0,1837	0,2267	0,2198	0,2795	–	< 0.0001	< 0.0001
**11**	0,1513	0,1116	0,1049	0,2357	0,1642	0,1244	0,1274	0,1453	0,1240	0,2170	–	< 0.0001
**12**	0,1088	0,1345	0,1125	0,1604	0,0888	0,1008	0,0925	0,1041	0,1332	0,1988	0,1158	–
**Right lateral**	**1**	**2**	**3**	**4**	**5**	**6**	**7**	**8**	**9**	**10**	**11**	**12**
**1**	–	0,0004*	< 0.0001	0,0002*	< 0.0001	< 0.0001	< 0.0001	< 0.0001	< 0.0001	< 0.0001	< 0.0001	< 0.0001
**2**	0,1702	–	0,0001*	0,0002*	0,0022*	< 0.0001	< 0.0001	< 0.0001	< 0.0001	< 0.0001	< 0.0001	0,0001*
**3**	0,1484	0,1284	–	< 0.0001	< 0.0001	< 0.0001	< 0.0001	0,0014*	< 0.0001	< 0.0001	< 0.0001	< 0.0001
**4**	0,1446	0,1594	0,1335	–	< 0.0001	< 0.0001	< 0.0001	< 0.0001	< 0.0001	< 0.0001	< 0.0001	< 0.0001
**5**	0,1724	0,2090	0,2036	0,1571	–	< 0.0001	< 0.0001	< 0.0001	0,0001*	0,0002*	< 0.0001	< 0.0001
**6**	0,1223	0,1120	0,1170	0,1120	0,1298	–	< 0.0001	< 0.0001	< 0.0001	< 0.0001	< 0.0001	< 0.0001
**7**	0,2139	0,2033	0,1053	0,1724	0,2260	0,1730	–	< 0.0001	< 0.0001	< 0.0001	< 0.0001	< 0.0001
**8**	0,1941	0,1853	0,0834	0,1667	0,2410	0,1676	0,0881	–	< 0.0001	< 0.0001	< 0.0001	< 0.0001
**9**	0,1777	0,1866	0,1059	0,1685	0,2231	0,1497	0,0794	0,0882	–	< 0.0001	< 0.0001	< 0.0001
**10**	0,1510	0,2067	0,1836	0,1542	0,1311	0,1521	0,2181	0,2152	0,2107	–	< 0.0001	< 0.0001
**11**	0,0960	0,1385	0,1219	0,1277	0,1714	0,0945	0,1790	0,1603	0,1418	0,1403	–	< 0.0001
**12**	0,1336	0,1875	0,1659	0,0930	0,1584	0,1286	0,1891	0,1847	0,1645	0,1533	0,1368	–

* statistically non-significant result

Table S8. Mahalanobis distances computed from canonical variate analysis (CVA) of the covariance matrices generated on averaged data for 122 gynostemia in *Epipactis helleborine* from different years of research.

**Dorsal**	**2017**	**2018**	**2019**
**2017**	–	< 0.0001	< 0.0001
**2018**	3.9683	–	< 0.0001
**2019**	2.3310	2.5707	–
**Frontal**	**2017**	**2018**	**2019**
**2017**	–	< 0.0001	< 0.0001
**2018**	3.8812	–	< 0.0001
**2019**	4.8799	3.4836	–
**Ventral**	**2017**	**2018**	**2019**
**2017**	–	< 0.0001	< 0.0001
**2018**	5.6024	–	< 0.0001
**2019**	5.1415	3.9017	–
**Right lateral**	**2017**	**2018**	**2019**
**2017**	–	< 0.0001	< 0.0001
**2018**	2.4670	–	< 0.0001
**2019**	2.6304	2.8883	–

Table S9. Procrustes distances computed from canonical variate analysis (CVA) of the covariance matrices generated on averaged data for 122 gynostemia in *Epipactis helleborine* from different years of research.

**Dorsal**	**2017**	**2018**	**2019**
**2017**	–	< 0.0001	0.0202*
**2018**	0.0795	–	0.0035*
**2019**	0.0502	0.0440	–
**Frontal**	2017	2018	2019
**2017**	–	0.0003*	< 0.0001
**2018**	0.0902	–	0.0002*
**2019**	0.1350	0.0772	–
**Ventral**	2017	2018	2019
**2017**	–	< 0.0001	0.0001*
**2018**	0.0934	–	0.0415*
**2019**	0.0899	0.0332	–
**Right lateral**	2017	2018	2019
**2017**	–	0.0075*	0.0078*
**2018**	0.0582	–	0.0250*
**2019**	0.0543	0.0448	–

* statistically non-significant result

Table S10. Descriptive statistics of all morphometric parameters of the gynostemium in *Epipactis helleborine* from different populations.

Continuous variables	Parameter	Kotowice	Milicz	Trestno	Żelazno
length [mm]	mean (standard deviation)	5.03 (0.24)	4.45 (0.28)	4.67 (0.34)	4.67 (0.40)
	median (interquartile range)	5.03 (4.86–5.18)	4.43 (4.28–4.57)	4.68 (4.44–4.86)	4.71 (4.36–4.87)
	min-max range	4.62–5.63	3.90–4.93	4.05–5.48	3.76–5.47
	coefficient of variation	4.82	6.26	7.20	8.62
width [mm]	mean (standard deviation)	2.87 (0.23)	2.65 (0.18)	2.86 (0.27)	3.10 (0.26)
	median (interquartile range)	2.83 (2.76–2.97)	2.68 (2.54–2.80)	2.81 (2.62–3.05)	3.13 (2.98–3.29)
	min-max range	2.50–3.35	2.22–2.91	2.38–3.39	2.40–3.64
	coefficient of variation	7.91	6.84	9.29	8.51
height [mm]	mean (standard deviation)	2.92 (0.36)	2.56 (0.23)	3.24 (0.33)	3.01 (0.37)
	median (interquartile range)	2.83 (2.63–3.12)	2.55 (2.34–2.71)	3.31 (3.08–3.47)	2.95 (2.70–3.27)
	min-max range	2.40–3.73	2.19–2.94	2.58–3.83	2.53–3.79
	coefficient of variation	12.28	9.08	10.11	12.36
stigma inclination angle [°]	mean (standard deviation)	64.46 (12.84)	72.01 (7.43)	47.44 (5.43)	69.28 (7.35)
	median (interquartile range)	62.40 (53.96–73.39)	73.08 (65.92–76.85)	46.95 (43.57–51.35)	68.64 (63.52–74.67)
	min-max range	45.21–95.32	56.31–87.46	36.93–60.49	53.40–81.68
	coefficient of variation	19.92	10.32	11.45	10.61

Table S11. Descriptive statistics of all morphometric parameters of the gynostemium in *Epipactis helleborine* from different ramets.

Continuous variables	Parameter	1	2	3	4	5	6	7	8	9	10	11	12
length [mm]	mean (standard deviation)	5.04 (0.20)	5.09 (0.17)	4.99 (0.32)	4.30 (0.20)	4.28 (0.20)	4.63 (0.26)	4.52 (0.25)	4.57 (0.28)	5.00 (0.30)	4.18 (0.26)	4.74 (0.20)	5.00 (0.34)
	median (interquartile range)	5.03 (4.98–5.10)	5.11 (5.04–5.22)	4.95 (4.79–5.02)	4.38 (4.20–4.42)	4.29 (4.19–4.44)	4.58 (4.43–4.89)	4.53 (4.32–4.70)	4.50 (4.40–4.83)	4.95 (4.80–5.20)	4.13 (4.05–4.31)	4.72 (4.66–4.83)	5.07 (4.77–5.26)
	min-max range	4.71-5.39	4.79-5.26	4.62-5.63	3.90-4.49	3.96-4.48	4.21-4.93	4.17-4.92	4.05-4.90	4.59-5.48	3.76-4.64	4.33-5.11	4.39-5.47
	coefficient of variation	3.96	3.40	6.44	4.55	4.63	5.55	5.61	6.04	6.09	6.13	4.18	6.82
width [mm]	mean (standard deviation)	2.87 (0.17)	2.83 (0.24)	2.89 (0.28)	2.51 (0.19)	2.66 (0.13)	2.74 (0.15)	2.74 (0.19)	2.73 (0.19)	3.19 (0.13)	2,8 (0,22)	3.13 (0.16)	3.30 (0.21)
	median (interquartile range)	2.84 (2.79-2.95)	2.81 (2.66-3.00)	2.86 (2.74-2.98)	2.57 (2.41-2.66)	2.66 (2.55-2.74)	2.80 (2.65-2.83)	2.76 (2.58-2.81)	2.67 (2.62-2.88)	3.20 (3.07-3.28)	2.81 (2.69-2.96)	3.13 (3.04 – 3.29)	3.29 (3.15-3.47)
	min-max range	2.58 - 3.17	2.55-3.15	2.50-3.35	2.22-2.68	2.53-2.86	2.43-2.91	2.51-3.07	2.38-3.12	2.99-3.39	2.40-3.13	2.79-3.38	3.02-3.64
	coefficient of variation	5.97	8.34	9.74	7.58	4.98	5.30	6.90	6.95	4.15	7.72	5.02	6.44
height [mm]	mean (standard deviation)	2.67 (0.18)	2.78 (0.26)	3.22 (0.33)	2.45 (0.18)	2.45 (0.24)	2.67 (0.21)	2.97 (0.32)	3.25 (0.27)	3.52 (0.14)	2.67 (0.10)	2.89 (0.17)	3.53 (0.18)
	median (interquartile range)	2.70 (2.55-2.82)	2.69 (2.59-2.96)	3.21 (3.05-3.33)	2.51 (2.32-2.57)	2.40 (2.26-2.56)	2.67 (2.55-2.86)	2.82 (2.68-3.26)	3.22 (3.13-3.41)	3.48 (3.45-3.57)	2.65 (2.63-2.76)	2.92 (2.77-3.01)	3.51 (3.43-3.67)
	min-max range	2.40-2.90	2.53-3.17	2.67-3.73	2.19-2.65	2.21-2.84	2.30-2.94	2.58-3.40	2.74-3.73	3.34-3.83	2.53-2.81	2.60-3.12	3.23-3.79
	coefficient of variation	6.68	9.37	10.27	7.23	10.01	8.02	10.81	8.27	3.90	3.58	5.76	5.14
stigma inclination angle [°]	mean (standard deviation)	77.15 (11.17)	63.22 (6.78)	54.00 (5.46)	69.26 (6.63)	74.36 (11.55)	72.62 (5.50)	46.84 (5.55)	47.09 (5.78)	48.66 (5.08)	64.44 (4.76)	75.32 (4.38)	63.97 (5.68)
	median (interquartile range)	78.34 (71.37-82.90)	63.42 (59.06-67.26)	53.12 (51.52-56.31)	68.44 (64.35-74.06)	77.56 (67.87-81.35)	74.51 (69.11-76.44)	47.22 (43.05-47.82)	46.49 (43.17-51.76)	46.98 (45.29-51.29)	63.44 (60.90-67.01)	76.02 (71.20-78.73)	65.81 (61.15-68.14)
	min-max range	61.78-95.32	53.93-72.42	45.21-63.69	60.05-78.51	56.31-87.46	63.86-83.38	40.64-60.49	36.93-55.13	42.68-59.67	59.14-74.08	68.20-81.68	53.40-70.10
	coefficient of variation	14.47	10.72	10.12	9.58	15.53	7.58	11.85	12.27	10.44	7.39	5.82	8.88

Table S12. Descriptive statistics of all morphometric parameters of the gynostemium in *Epipactis helleborine* from different years of research.

Continuous variables	Parameter	2017	2018	2019
length [mm]	mean (standard deviation)	4.50 (0.39)	4.66 (0.32)	4.88 (0.34)
	median (interquartile range)	4.42 (4.22–4.75)	4.71 (4.45–4.85)	4.84 (4.64–5.10)
	min-max range	3.76–5.39	3.96–5.26	4.21–5.63
	coefficient of variation	8.67	6.78	6.95
width [mm]	mean (standard deviation)	2.73 (0.23)	2.88 (0.26)	3.01 (0.30)
	median (interquartile range)	2.73 (2.59–2.88)	2.87 (2.64–3.09)	3.00 (2.81–3.28)
	min-max range	2.22–3.17	2.38–3.38	2.43–3.64
	coefficient of variation	8.24	9.08	9.93
height [mm]	mean (standard deviation)	2.71 (0.28)	2.94 (0.35)	3.19 (0.43)
	median (interquartile range)	2.66 (2.56–2.81)	2.96 (2.69–3.17)	3.32 (2.86–3.52)
	min-max range	2.19–3.40	2.21–3.73	2.30–3.83
	coefficient of variation	10.47	11.97	13.46
stigma inclination angle [°]	mean (standard deviation)	62.96 (13.60)	63.28 (14.41)	60.86 (10.97)
	median (interquartile range)	63.35 (50.56–73.33)	67.04 (51.71–75.46)	61.88 (51.73–68.91)
	min-max range	40.64–95.32	36.93–87.46	42.68–83.38
	coefficient of variation	21.61	22.78	18.02

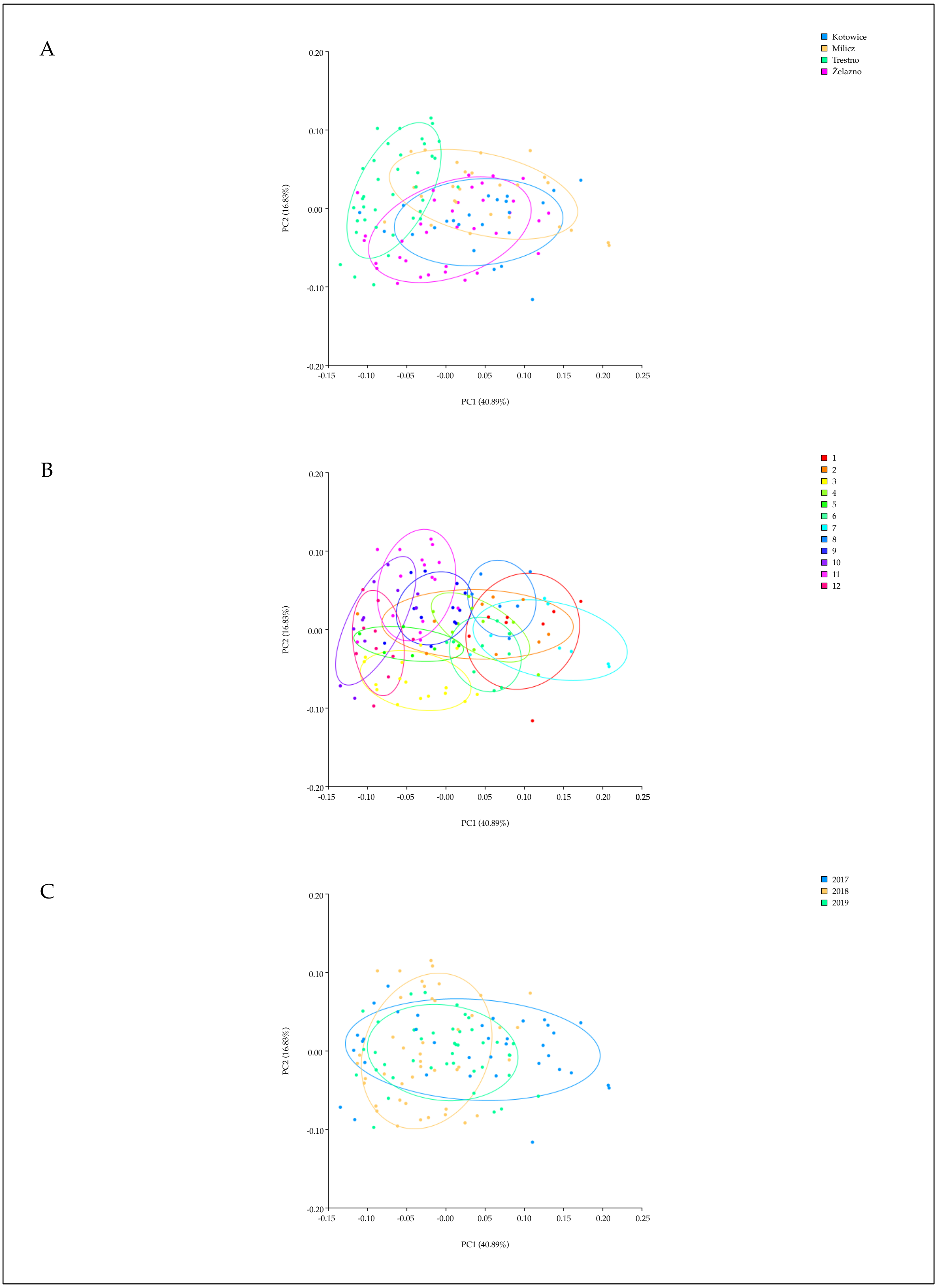

Figure S1. Plot of the first two principal components (PCs) from principal component analysis for 122 gynostemium configurations in dorsal view, showing results for different populations (**A**), ramets (**B**), and years of research (**C**).

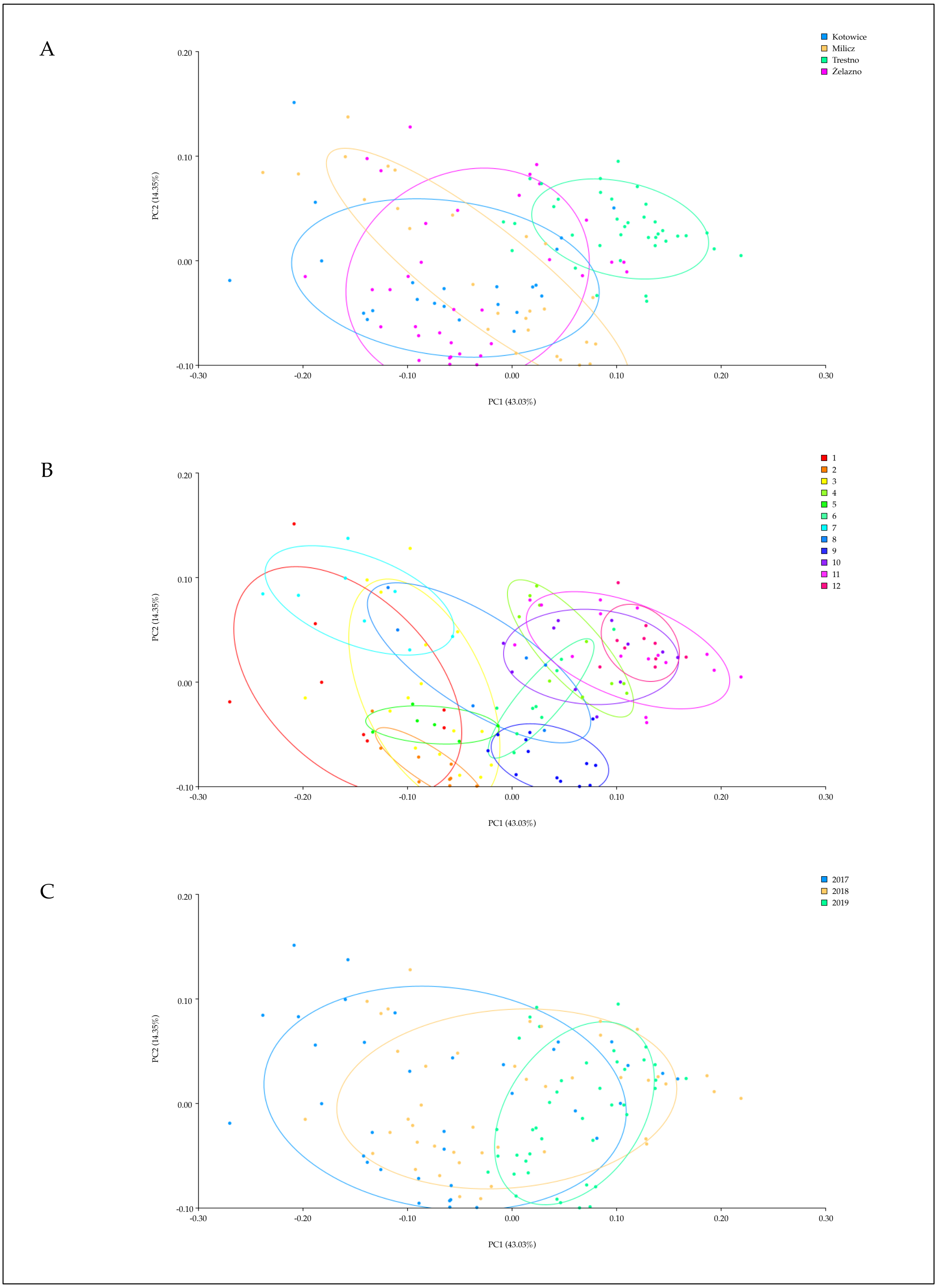

Figure S2. Plot of the first two principal components (PCs) from principal component analysis for 122 gynostemium configurations in frontal view, showing results for different populations (**A**), ramets (**B**), and years of research (**C**).

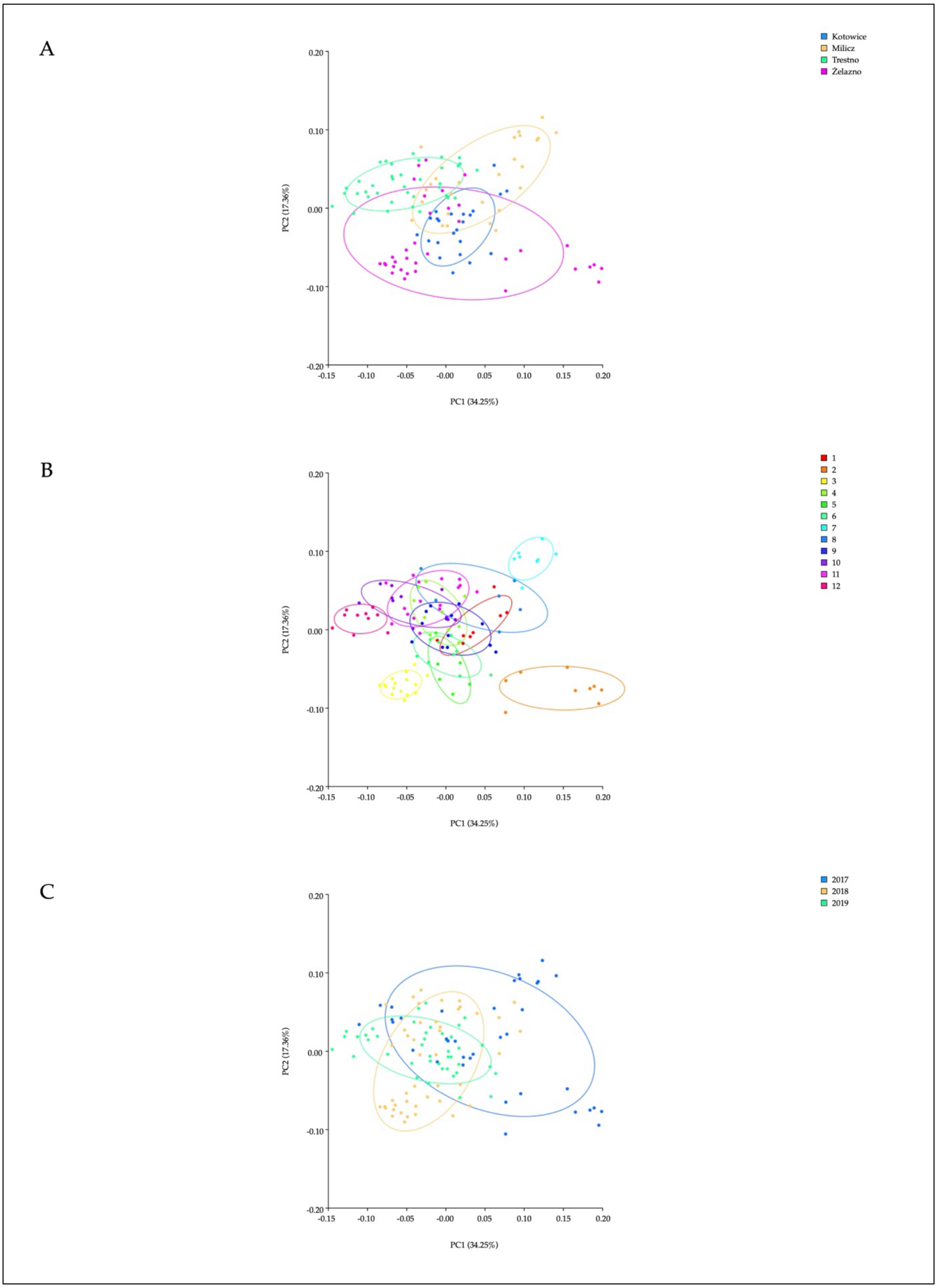

Figure S3. Plot of the first two principal components (PCs) from principal component analysis for 122 gynostemium configurations in ventral view, showing results for different populations (**A**), ramets (**B**), and years of research (**C**).

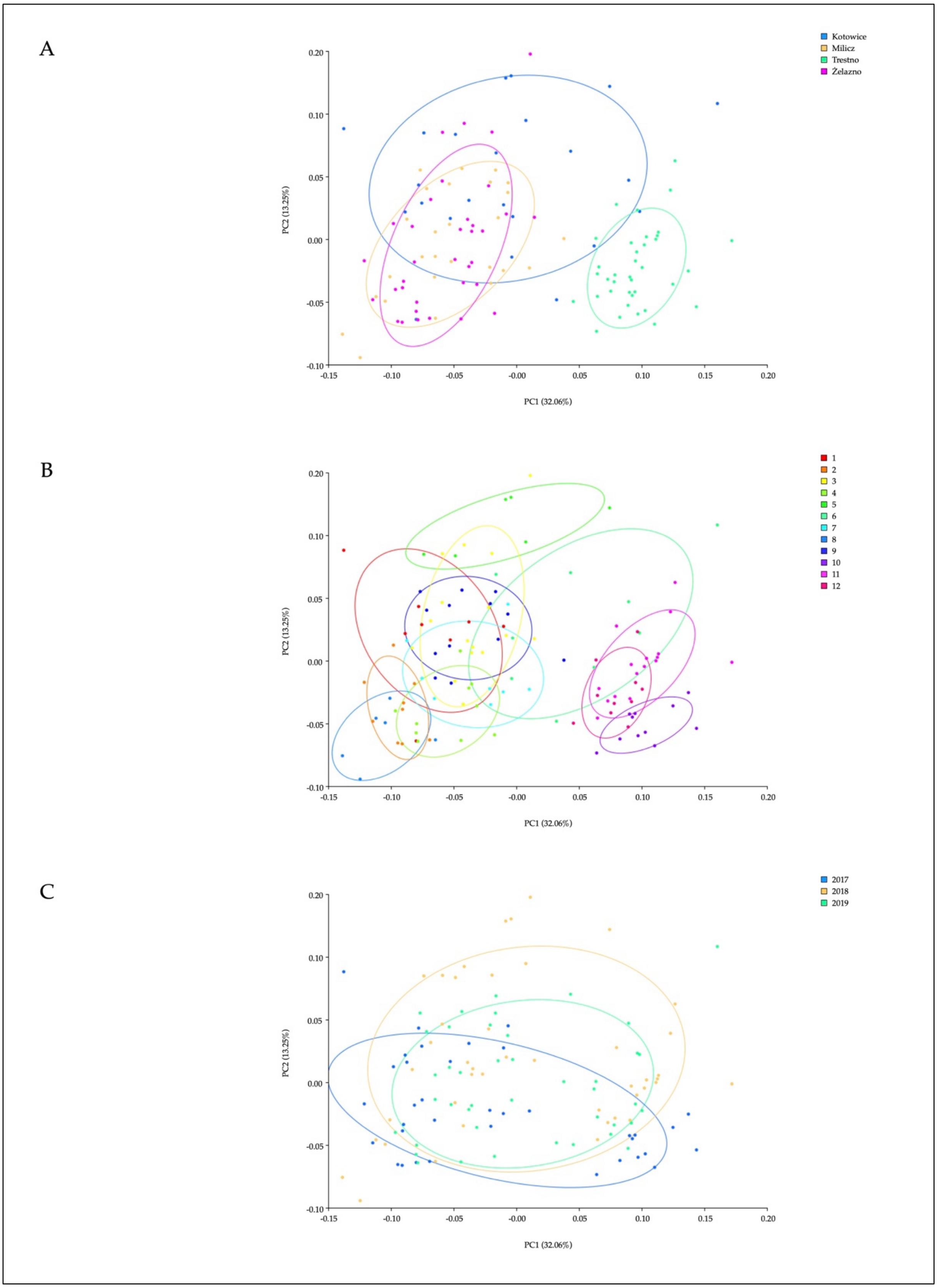

Figure S4. Plot of the first two principal components (PCs) from principal component analysis for 122 gynostemium configurations in right lateral view, showing results for different populations (**A**), ramets (**B**), and years of research (**C**).

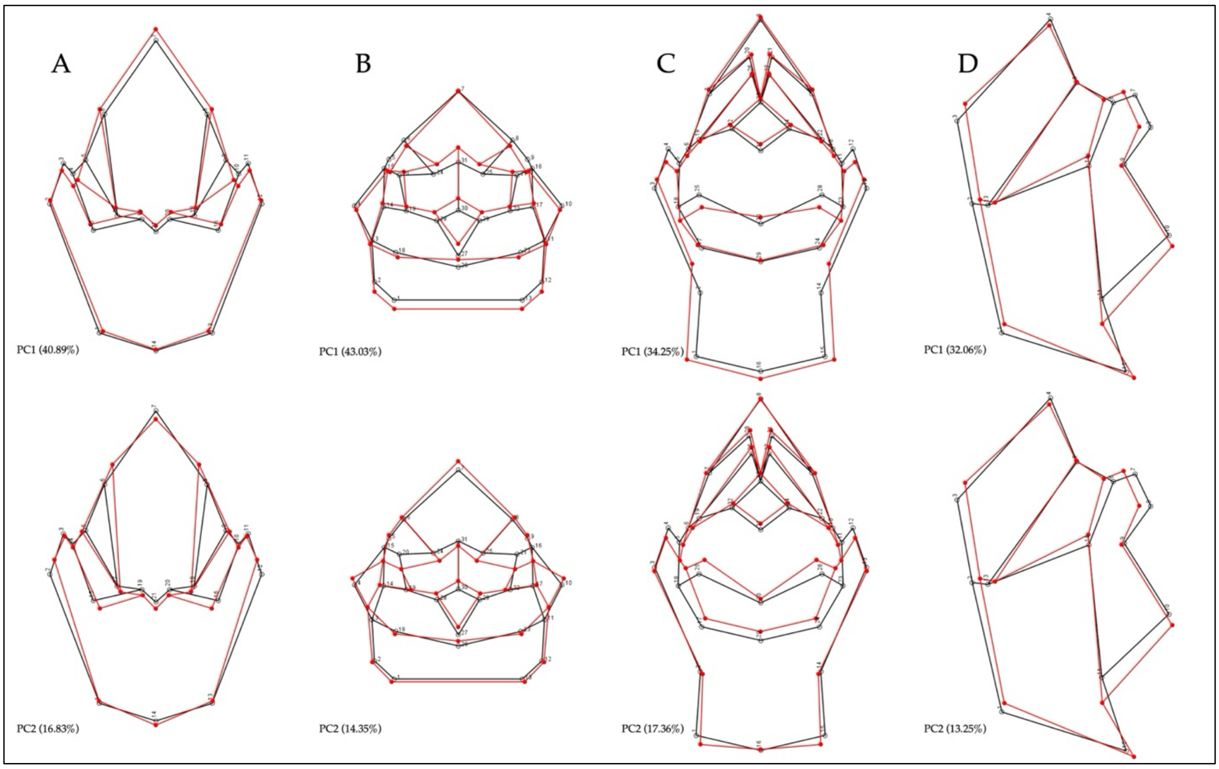

Figure S5. Wireframe graphs describing gynostemium shape changes between the minimum and maximum values of PC1 and PC2, showing results for the dorsal (**A**), frontal (**B**), ventral (**C**), and right lateral (**D**) views.

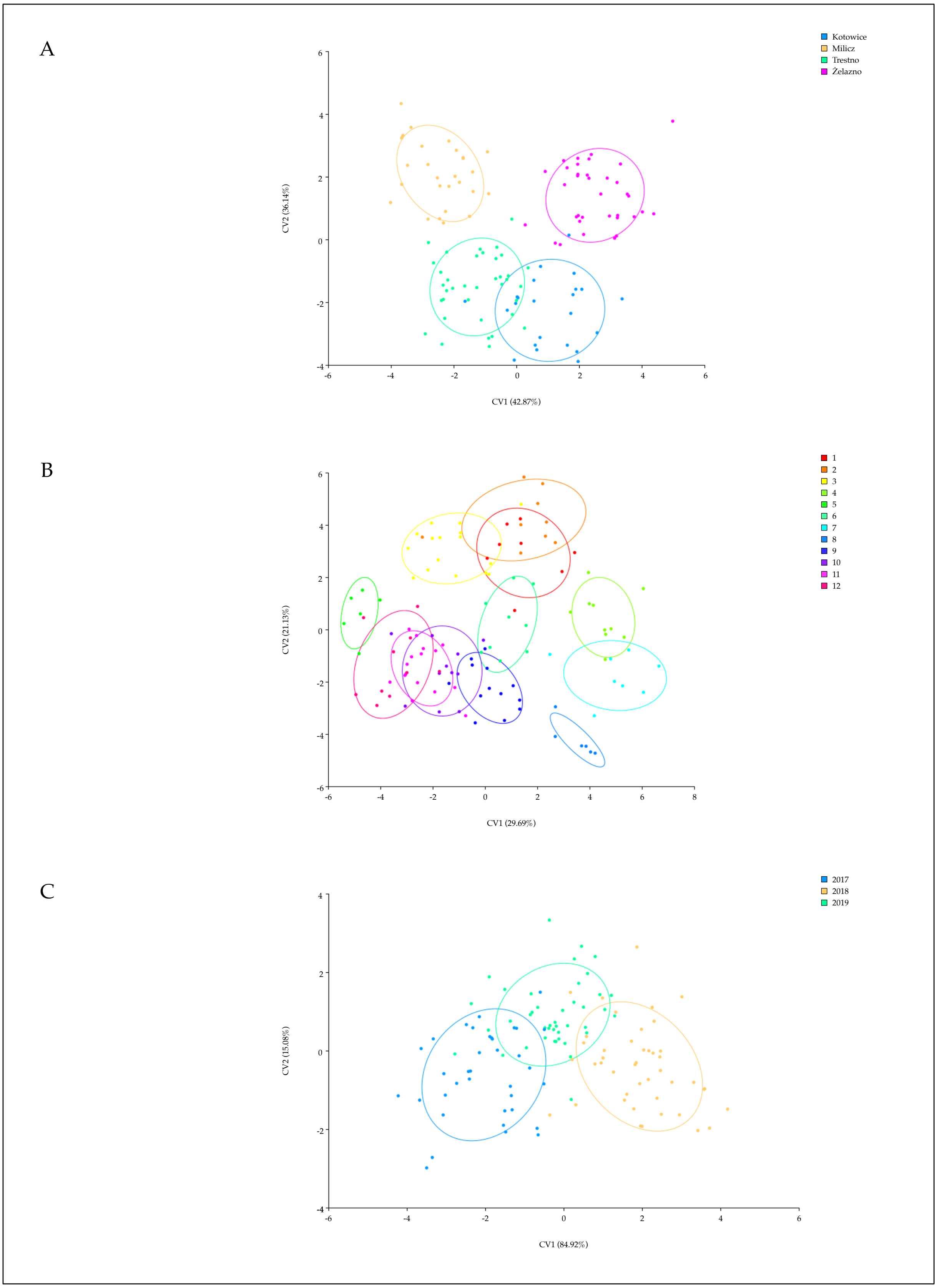

Figure S6. Plot of the first two canonical variates (CVs) from canonical variate analysis conducted on averaged data for 122 gynostemia in dorsal view, showing results for different populations (**A**), ramets (**B**), and years of research (**C**).

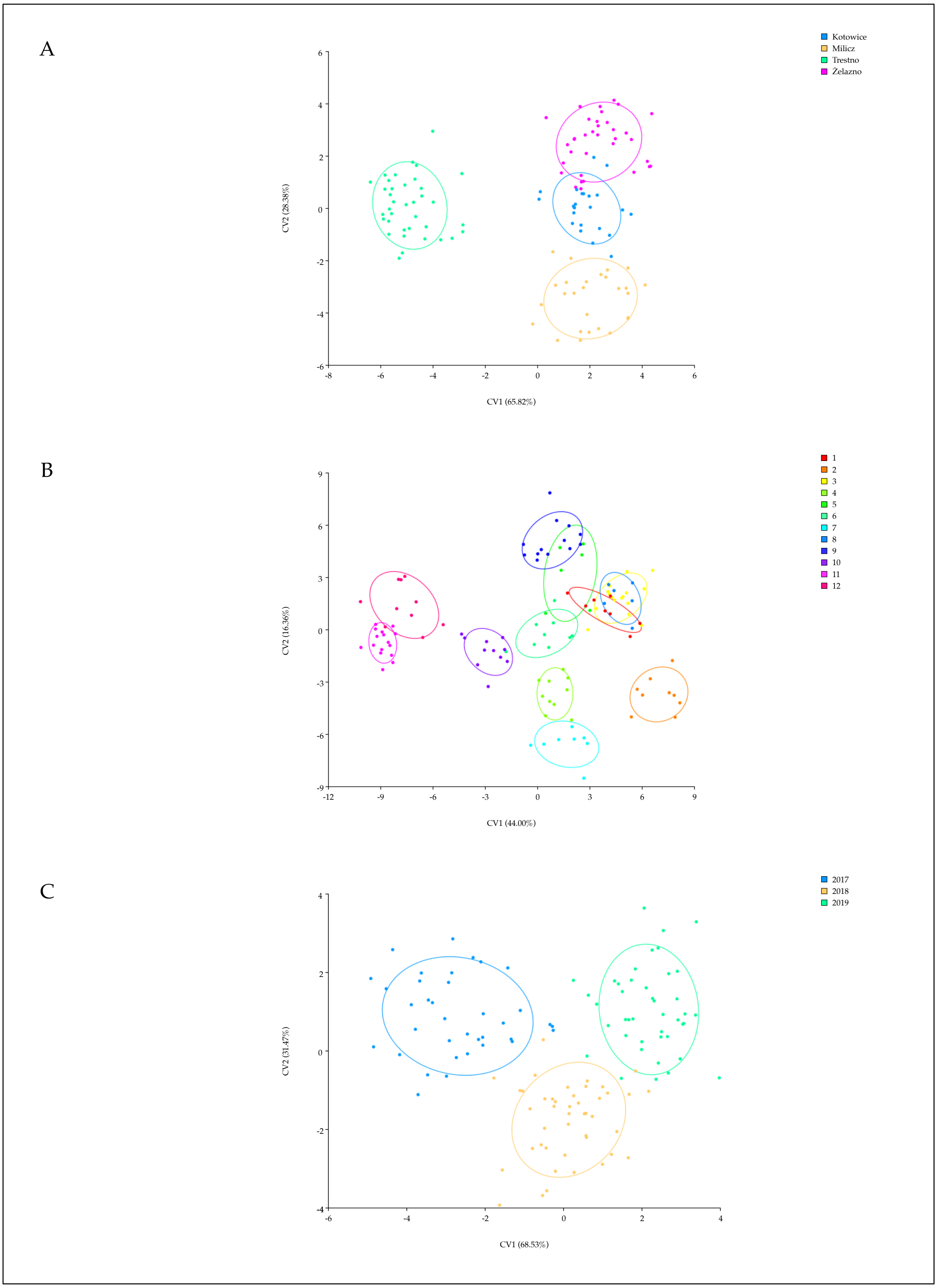

Figure S7. Plot of the first two canonical variates (CVs) from canonical variate analysis conducted on averaged data for 122 gynostemia in frontal view, showing results for different populations (**A**), ramets (**B**), and years of research (**C**).

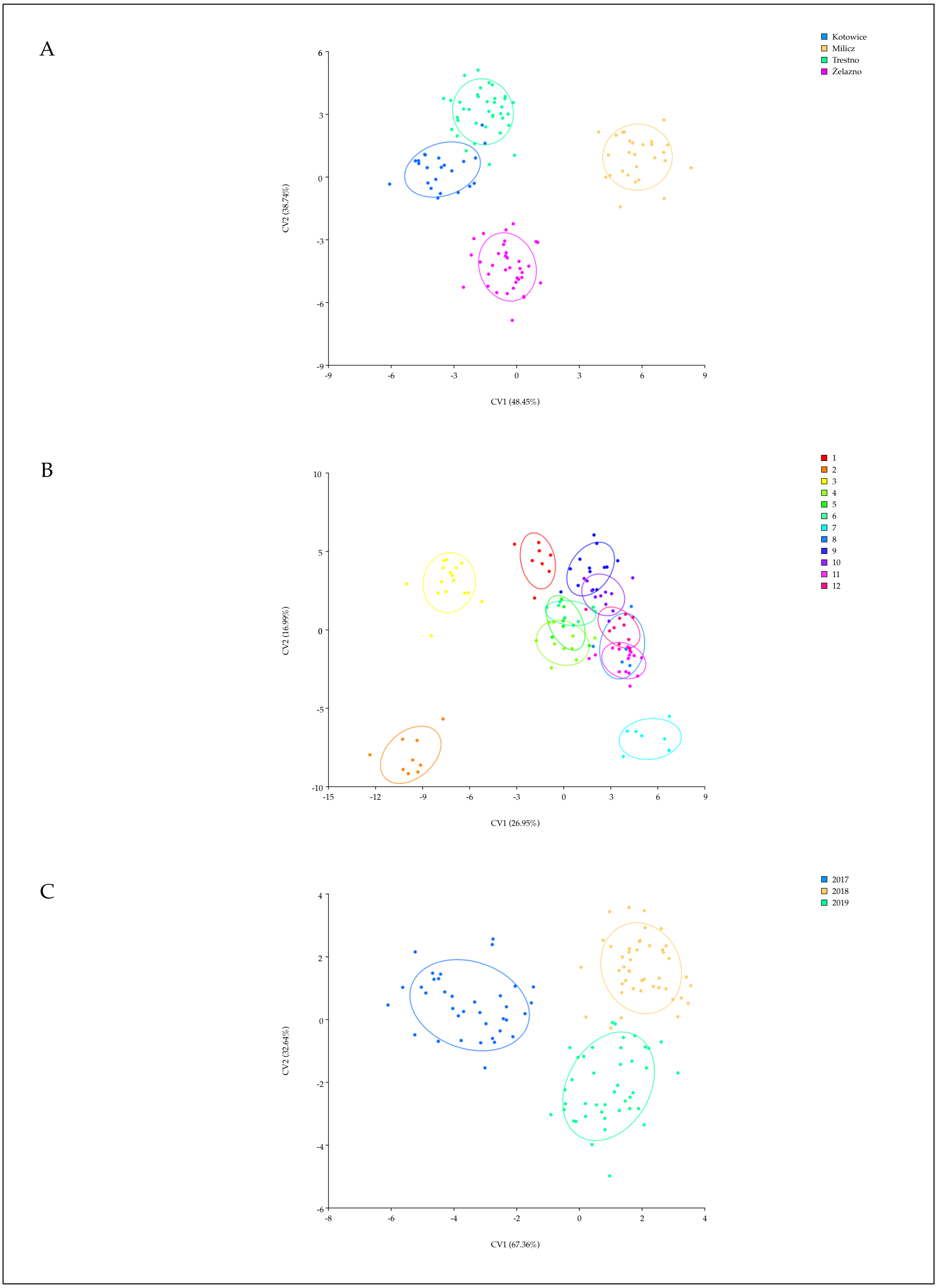

Figure S8. Plot of the first two canonical variates (CVs) from canonical variate analysis conducted on averaged data for 122 gynostemia in ventral view, showing results for different populations (**A**), ramets (**B**), and years of research (**C**).

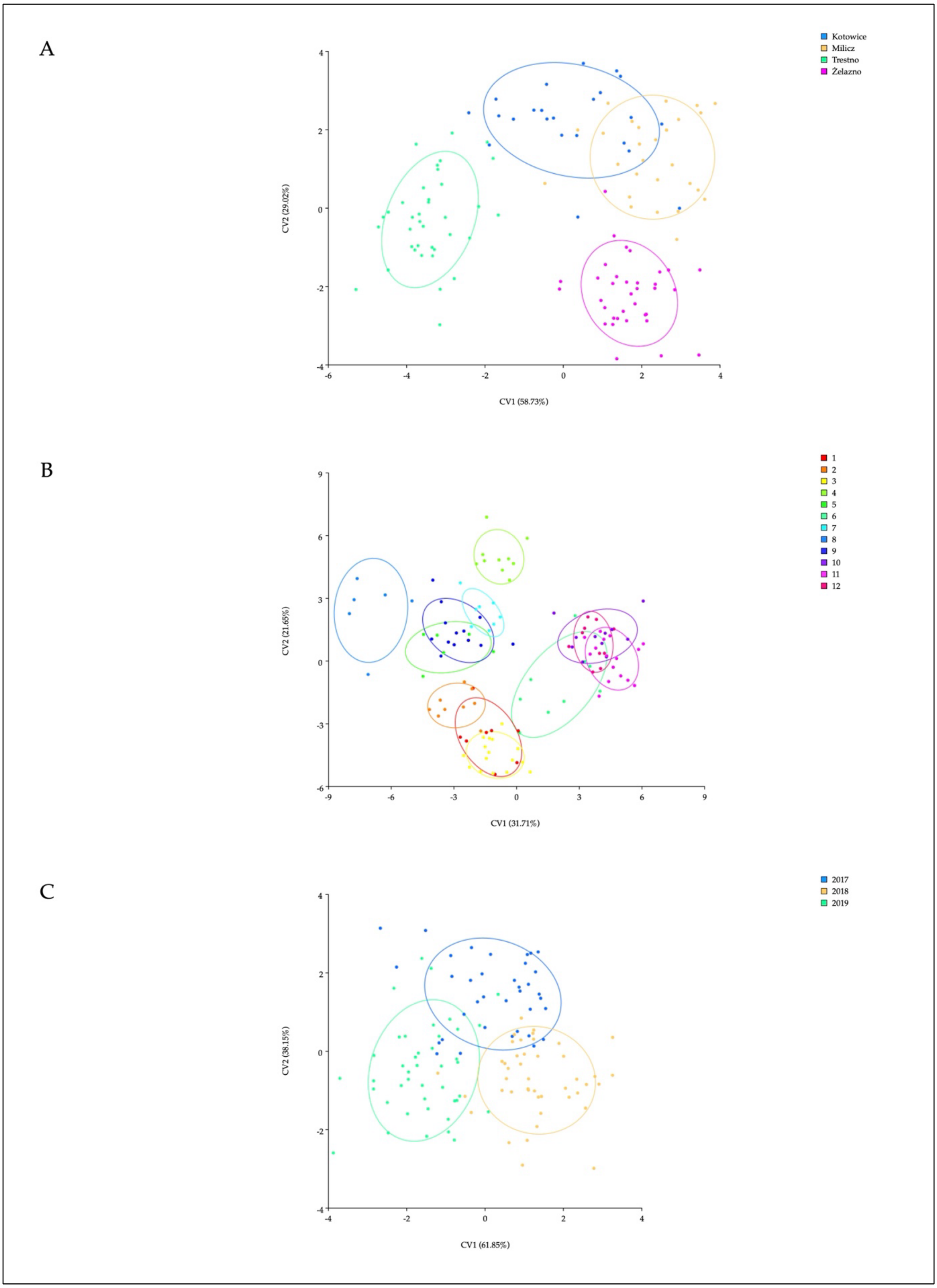

Figure S9. Plot of the first two canonical variates (CVs) from canonical variate analysis conducted on averaged data for 122 gynostemia in right lateral view, showing results for different populations (**A**), ramets (**B**), and years of research (**C**).

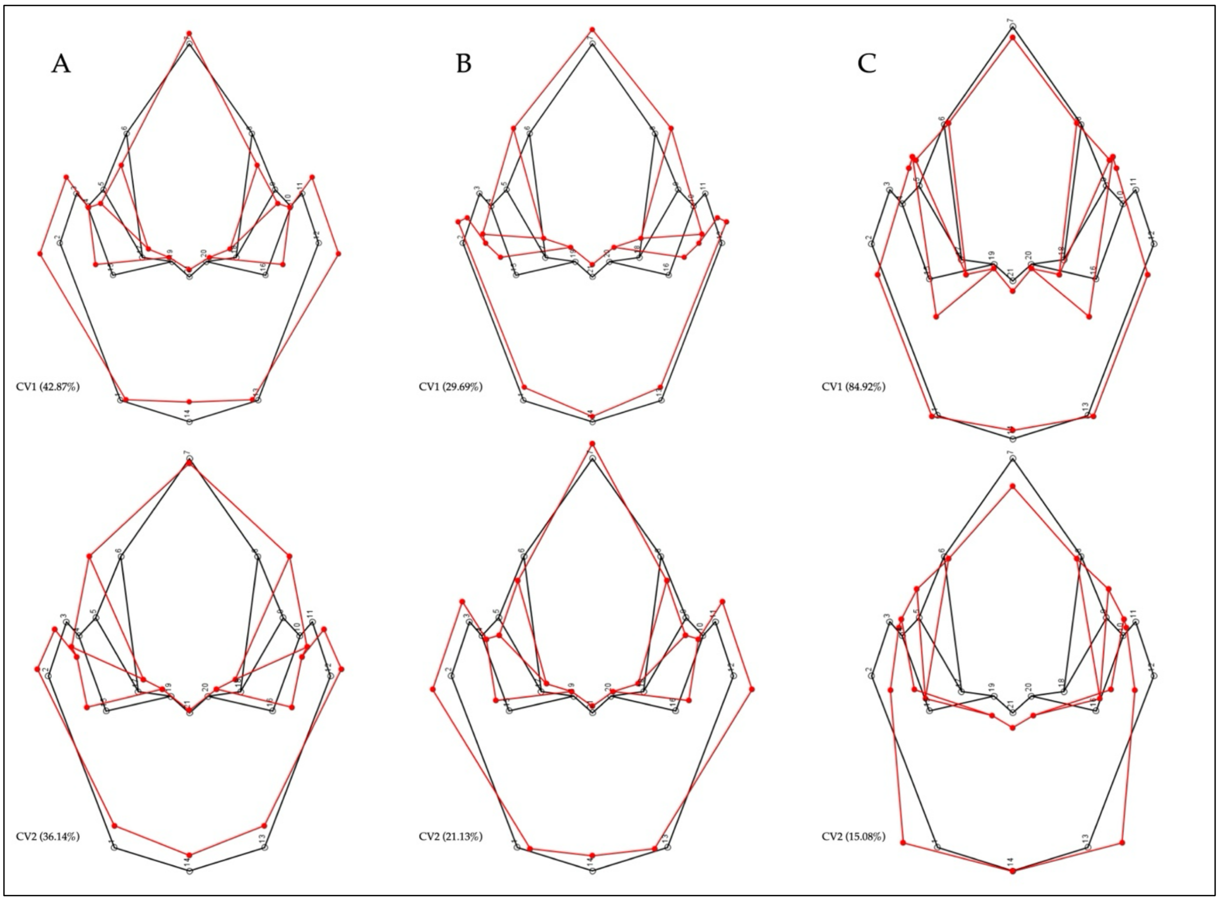

Figure S10. Wireframe graphs describing gynostemium shape changes in dorsal view between the minimum and maximum values of CV1 and CV2, showing results for different populations (**A**), ramets (**B**), and years of research (**C**).

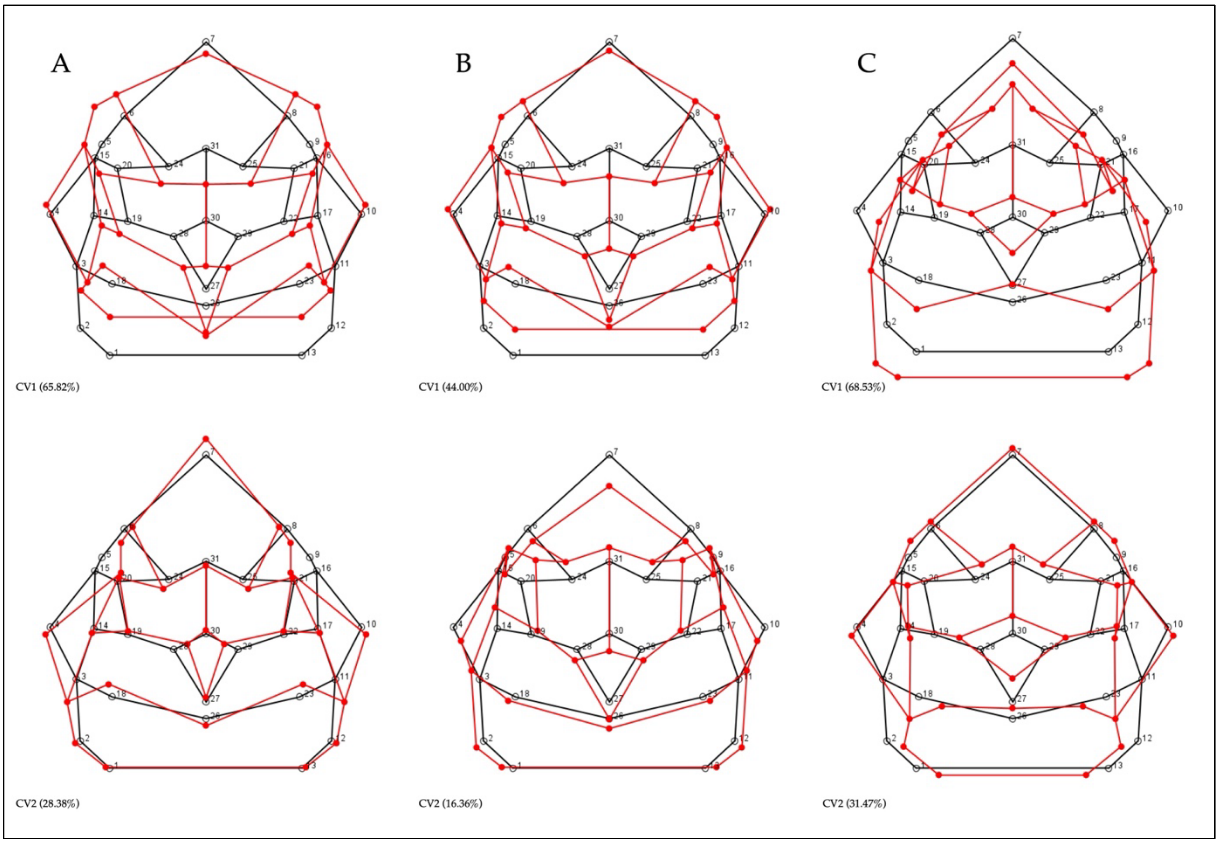

Figure S11. Wireframe graphs describing gynostemium shape changes in frontal view between the minimum and maximum values of CV1 and CV2, showing results for different populations (**A**), ramets (**B**), and years of research (**C**).

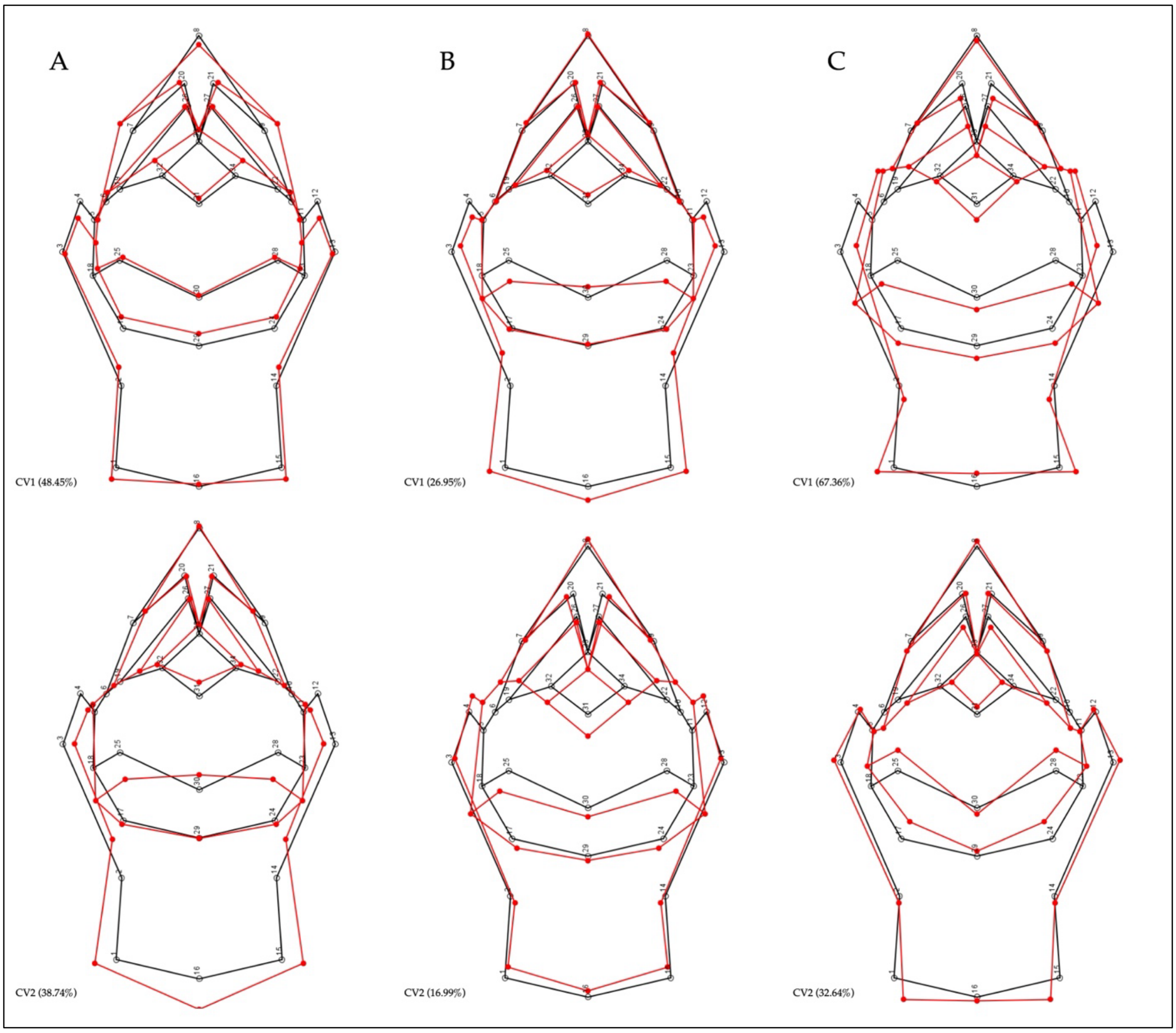

Figure S12. Wireframe graphs describing gynostemium shape changes in ventral view between the minimum and maximum values of CV1 and CV2, showing results for different populations (**A**), ramets (**B**), and years of research (**C**).

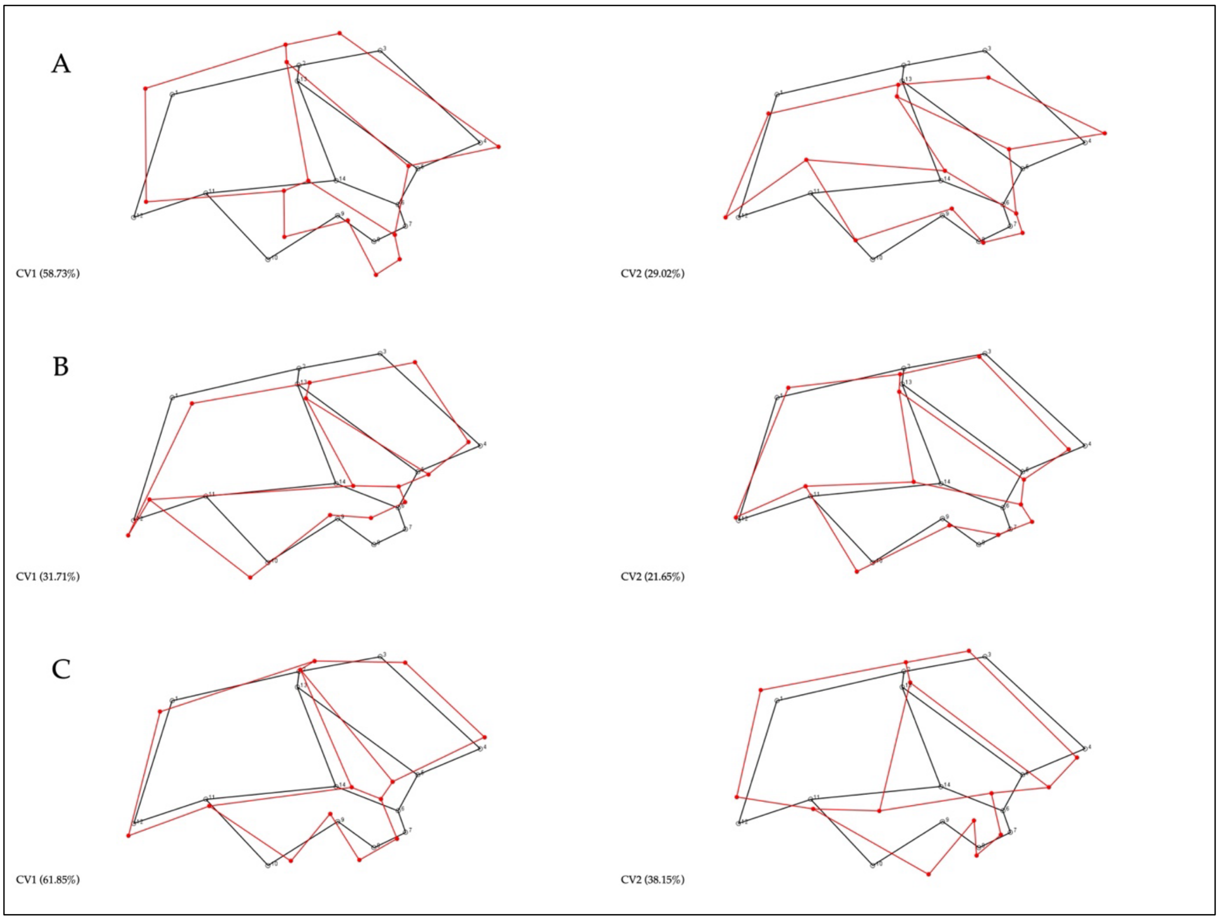

Figure S13. Wireframe graphs describing gynostemium shape changes in right lateral view between the minimum and maximum values of CV1 and CV2, showing results for different populations (**A**), ramets (**B**), and years of research (**C**).
